# A Guided, Internet-Based Stress Management Intervention for University Students With High Levels of Stress: Feasibility and Acceptability Study

**DOI:** 10.2196/45725

**Published:** 2023-11-10

**Authors:** Yagmur Amanvermez, Eirini Karyotaki, Pim Cuijpers, Marketa Ciharova, Marianne Donker, Petra Hurks, Elske Salemink, Philip Spinhoven, Sascha Struijs, Leonore M de Wit

**Affiliations:** 1 Department of Clinical, Neuro and Developmental Psychology Amsterdam Public Health Research Institute Vrije Universiteit Amsterdam Netherlands; 2 Department of Medical and Clinical Psychology Tilburg University Tilburg Netherlands; 3 Black Dog Institute, University of New South Wales Sydney Australia; 4 Department of Health Sciences Vrije Universiteit Amsterdam Amsterdam Netherlands; 5 Faculty of Psychology and Neuroscience Maastricht University Maastricht Netherlands; 6 Department of Clinical Psychology Utrecht University Utrecht Netherlands; 7 Institute of Psychology Leiden University Leiden Netherlands

**Keywords:** internet-based interventions, stress management, university students, e–mental health, feasibility study, stress, digital interventions

## Abstract

**Background:**

Transitioning to adulthood and challenges in university life can result in increased stress levels among university students. Chronic and severe stress is associated with deleterious psychological and physiological effects. Digital interventions could succeed in approaching and helping university students who might be at risk; however, the experiences of students with internet-based stress management interventions are insufficiently understood.

**Objective:**

This study aims to explore the feasibility; acceptability; and changes in perceived stress, depressive symptoms, and quality of life from baseline to posttest assessment of a 5-session, internet-based stress management intervention guided by an e-coach, developed for university students experiencing high levels of stress.

**Methods:**

A single-arm study was conducted. Students were recruited from different channels, mainly from a web survey. Students were eligible if they (1) scored ≥20 on the Perceived Stress Scale–10, (2) were aged ≥18 years, and (3) were studying at one of the participating universities. Feasibility and acceptability of the intervention were investigated using several indications, including satisfaction (Client Satisfaction Questionnaire–8) and usability (System Usability Scale–10). We also investigated the indicators of intervention adherence using use metrics (eg, the number of completed sessions). Our secondary goal was to explore the changes in perceived stress (Perceived Stress Scale–10), depressive symptoms (Patient Health Questionnaire–9), and quality of life (EQ-5D-5L scale) from baseline to posttest assessment. In addition, we conducted semistructured interviews with intervention completers and noncompleters to understand user experiences in depth. For all primary outcomes, descriptive statistics were calculated. Changes from baseline to posttest assessment were examined using 2-tailed paired sample *t* tests or the Wilcoxon signed rank test. Qualitative data were analyzed using thematic analysis.

**Results:**

Of 436 eligible students, 307 (70.4%) students started using the intervention. Overall, 25.7% (79/307) completed the core sessions (ie, sessions 1-3) and posttest assessment. A substantial proportion of the students (228/307, 74.3%) did not complete the core sessions or the posttest assessment. Students who completed the core sessions reported high satisfaction (mean 25.78, SD 3.30) and high usability of the intervention (mean 86.01, SD 10.25). Moreover, this group showed large reductions in perceived stress (Cohen *d*=0.80) and moderate improvements in depression score (Cohen *d*=0.47) and quality of life (Cohen *d*=−0.35) from baseline to posttest assessment. Qualitative findings highlight that several personal and intervention-related factors play a role in user experience.

**Conclusions:**

The internet-based stress management intervention seems to be feasible, acceptable, and possibly effective for some university students with elevated stress levels. However, given the high dropout rate and qualitative findings, several adjustments in the content and features of the intervention are needed to maximize the user experience and the impact of the intervention.

**Trial Registration:**

Netherlands Trial Register 8686; https://onderzoekmetmensen.nl/nl/trial/20889

**International Registered Report Identifier (IRRID):**

RR2-10.1016/j.invent.2021.100369

## Introduction

### Background

In general, university students, typically aged between 18 and 22 years, experience a developmental transition from adolescence to adulthood. This transition is characterized by ongoing self-exploration, identity formation, managing increased autonomy, and new responsibilities. This period is critical because the onset of common mental disorders generally occurs during adolescence and young adulthood [1]. Aside from these developmental transitions, academic, financial, personal, or relationship issues can be sources of difficulties in university life [2-4]. Not surprisingly, psychological stress is widespread among university students [5,6].

Studies about the mental health of university students show a link between high levels of perceived stress and impaired academic performance [7], increased levels of depressive and anxiety symptoms [8], sleep problems [9], alcohol consumption [10], and somatic complaints [11]. Ongoing excessive stress might contribute to the progression of psychological problems [1] and pave the way for mental disorders and physical disorders, such as asthma or cardiovascular diseases, in the long run [12]. Given the individual and societal impact (eg, economic cost) [13,14], providing preventive psychological interventions to reduce the stress levels for university students is of utmost importance. Such interventions often focus on improving the so-called stress management skills, which are defined as psychological, physiological, and behavioral strategies to cope with stressful situations and alleviate the negative impact of stress [15,16].

Previous reviews show that university students can benefit from stress management interventions [17-19]. However, having a busy schedule, preference to deal with their problems independently, lack of anonymity, and long waiting lists for professional mental help could result in low intervention uptake [20,21]. Internet-based interventions might counteract some of these barriers owing to their potential to increase accessibility and overcome stigma for help seeking [22,23].

Although internet-based interventions appear to be an alternative to face-to-face interventions for university students [24,25], the small effects of internet-based interventions on stress in this population [26] and the low adherence raise some questions about these interventions [24,27,28]. Previous studies investigating internet-based interventions for university students reported that dropout rates ranged from 22% to 65% [29,30]. There is also evidence suggesting a significant association between the young age of the participants and low adherence to internet-based interventions [28,31]. Certain characteristics of university students such as encountering more frequent changes in life conditions than the general population can plausibly influence their experiences with such interventions [29]. Personal (eg, busy schedule and lack of motivation) or intervention-related reasons (eg, technical problems and familiarity with the content) could influence the use of such interventions in young populations [32,33]; however, a deep understanding of university students’ unique experiences with internet-based interventions is particularly needed. Although studies investigating the effects of stress management interventions alongside universal and indicated interventions exist for higher education students [34-37], only a few studies have investigated the effects of such interventions specifically among students with high stress levels using validated tools. In a recent meta-analysis, only 4 studies investigating digital stress management interventions for students with high levels of stress could be retrieved [34]. Moreover, cocreated interventions involving university students are limited. Developing targeted interventions, particularly for students experiencing high levels of stress is crucial to mitigate the abovementioned profound impact of stress on mental health and well-being.

Feasibility studies are recommended to set out the implementation of the study as planned, and users’ responses to the novel intervention should be examined before conducting randomized controlled trials (RCTs) [38,39]. One of the key indicators of feasibility is acceptability, which could be conceptualized by various parameters, including satisfaction, usability, adherence, and effectiveness [38]. Satisfaction reflects the users’ evaluations of whether their expectations and needs regarding the intervention are met [38,40]. Usability refers to the extent to which users find the intervention to be user-friendly and easy to navigate [41]. Adherence is conceptualized by the actual use of the intervention and how users engage with it [42]. Other indicators of feasibility are the helpfulness of the intervention and positive intervention outcomes on the symptom of interest (ie, effectiveness) [38,39]. These constructs are associated with each other and could contribute to the increased impact of the intervention. For example, usability could be associated with high satisfaction, which results in intervention adherence leading to large effects of the intervention and vice versa [43-45].

Feasibility studies can help us to understand the users’ considerations and evaluations in great detail [38,46]. In addition to objective metrics such as satisfaction scales or tracking time spent and completed sessions, qualitative data are valuable in understanding users’ subjective experiences with the intervention. They can offer in-depth information about which parts of the intervention were particularly useful or engaging. Incorporating qualitative data into research appears to be an important avenue, as it helps in designing interventions that are user-friendly, relevant, and appealing [27,42,47-49].

### Objective

To address the abovementioned gaps, we performed a feasibility study in which we identified the students’ experiences with a guided, internet-based stress management intervention regarding feasibility and acceptability using objective metrics and qualitative data. The second objective of this study was to investigate whether students, who completed the intervention, showed improvement in perceived stress, reduction in depression, and increase in quality of life from baseline to posttest assessment.

## Methods

### Study Design

This open trial was conducted as a part of the Caring Universities project, the Dutch branch of the World Health Organization World Mental Health International College Student (WMH-ICS) initiative. Details about WMH-ICS are reported elsewhere [50]. We designed a single-arm study to assess the feasibility, acceptability, adherence, and preliminary evidence of the effectiveness of a guided, internet-based stress management intervention on stress, depression, and quality of life for university students with high levels of stress. This study was preregistered in the Netherlands Trial Register (8686), and the study protocol has been published elsewhere [51]. The changes to protocol are reported in Multimedia Appendix 1.

The study followed the CONSORT-EHEALTH (Consolidated Standards of Reporting Trials of Electronic and Mobile Health Applications and Online Telehealth) guidelines (version 1.6.1) [52] (Multimedia Appendix 2).

The study was originally planned to be conducted at 4 universities in the Netherlands: Vrije University Amsterdam, Leiden University, Maastricht University, and Utrecht University. During the study, Erasmus University Rotterdam, University of Amsterdam, and InHolland University of Applied Sciences also joined the project.

### Ethical Considerations

Ethics approval was obtained from the Scientific and Ethical Review Board of Vrije University Amsterdam (2020.088), and the ethical committees of all other participating universities adhered to the same guidelines. Before data collection, participants provided written consent after receiving information about the study. We anonymized the participants using unique ID numbers for each individual. Participants were not compensated for their participation in the survey or following the intervention; however, we provided a voucher worth €25 (US $27.36) to those who participated in the interviews. Participation in this intervention was entirely voluntary, and they could discontinue the study at any time they want.

### Eligibility Criteria

The eligibility criteria for participants were as follows: (1) aged ≥18 years, (2) enrollment in one of the participating universities or universities of applied sciences in the Caring Universities project, and (3) having elevated levels of perceived stress (Perceived Stress Scale–10 [PSS-10] score ≥20) [53]. This cutoff score was predefined by calculating an SD (SD 6.2) above the average score (mean 14.2) based on normative data from the sample aged 18 to 29 years [54].

Students were excluded if they: (1) scored ≥20 on the Patient Health Questionnaire–9 (PHQ-9) [55]; (2) had an indication of suicidality, which is defined as having a score >2 on the PHQ-9 item 9 and having a score ≥1 in response to the question “In approximately how many months during the past 12 months did you think about how you might kill yourself or work out a plan of how to kill yourself?” along with a response of “somewhat likely” to “very likely” for the question “how likely do you think it is that you will act on this plan in the next 12 months?”; (3) did not provide informed consent; and (4) provided incomplete data at the baseline assessment.

### Recruitment

Several recruitment strategies were planned to reach students. The main recruitment method was the survey designed by WMH-ICS, which is a standard annual survey offered to all students enrolled in the institutes involved in Caring Universities. As a standard option in this survey, students can opt to receive feedback based on their screening results, and if they opted in, they were advised to follow the intervention if they had elevated stress levels. In addition, a website was created and shared with all students enrolled in the institutes mentioned previously. We also made some announcements at several locations on campuses and university websites. Student psychologists, student advisers, and mentors also recommended interventions to students who might need help.

### Intervention

In this study, the internet-based stress management intervention called Rel@x was developed based on the principles of cognitive behavioral therapy (CBT) and the transactional model of stress [56]. This intervention was cocreated with university students through several focus groups to inquire about their experiences with stress and their preferences for a web-based stress management intervention that suited their needs.

It consisted of 5 main sessions, each designed to last approximately 60 minutes on a weekly basis. However, students were allowed to follow at their own pace and frequency based on their preferences. The intervention started with a general introduction about how to use the platform and by setting expectations for Rel@x. Following the introduction session, students continued the sessions in a predefined order. The sessions covered the following topics, respectively: (1) psychoeducation about stress, (2) coping skills and emotion regulation, (3) cognitive restructuring, (4) problem-solving strategies, and (5) reviewing the stress responses and setting future goals. Each session was built upon the previous one; therefore, to continue to the next session, participants must have completed the previous session. The core sessions were defined based on the theoretical foundations of the intervention (ie, CBT and the transactional model of stress) and previous studies [56-58]. The theoretical background of the intervention suggests that identifying cognitive appraisals of the stressor and one’s coping skills and disputing maladaptive thoughts around them lead to symptom change. The information and strategies related to these mechanisms were mainly provided in the first 3 sessions. Optional sessions were provided covering more specific topics related to university life including adapting to a new culture, assertiveness, time management and procrastination, and building a healthy lifestyle. Students could choose their own pace for completing the sessions; however, completing each session weekly was recommended to process the information and complete the assignments provided at the end of each session.

A stress diary and stress tracker in which participants can rate their stress levels using emoticons were embedded for monitoring the stress levels periodically as add-on features to the intervention. Screenshots of the intervention can be found in Multimedia Appendix 3.

### Guidance

The e-coaches providing guidance for the intervention were trained (research) master’s students in clinical psychology and third-year clinical psychology Bachelor’s students at the end of the second semester at Vrije Universiteit Amsterdam. The qualifications and selection procedure of the e-coaches are explained in detail in the protocol paper [51]. Each participant was able to choose their own e-coach based on their profiles. Participants could also choose to be anonymous to their e-coach if they preferred. After completing each session, participants received written, personalized feedback from the e-coach within 3 to 5 days. The e-coaches provided motivational feedback to facilitate adherence, but they were not instructed to deliver any CBT-related therapeutic content. We provided training to the e-coaches and monitored their performance during the supervision meetings to maintain a clear distinction between their clinical knowledge and their role as an e-coach. In addition to guidance from the e-coach, participants who were inactive in the intervention for 2 weeks received an automated email to their provided email address as a reminder to increase adherence.

### Outcome Measures

#### Primary Outcomes

In this study, the primary outcomes were satisfaction with the intervention and usability (ie, the extent to which the intervention is perceived as easy to use in terms of instructions and learnability of the system). The Client Satisfaction Questionnaire–8 (CSQ-8) was used to measure the level of satisfaction [59]. It includes 8 questions regarding the overall evaluations of the intervention. The questions are rated on a 4-point Likert scale (with a sum score range from 8-32), and high scores correspond to high satisfaction. CSQ-8 has demonstrated good psychometric characteristics [45].

The System Usability Scale (SUS-10) was applied to assess usability [60]. It is a 10-item self-report scale (sum score ranges from 0-100), and high scores represent great usability of the intervention. It has high validity and reliability [61].

Regarding adherence, we calculated the number of students who registered for the intervention and continued the core sessions of the intervention. In this study, completion of at least 3 sessions out of 5 was deemed to be sufficient to expose the core elements of the intervention, and that is why we refer to these as “core sessions” as explained previously. Therefore, students who discontinued the intervention within the first 3 sessions were classified as noncompleters.

#### Secondary Outcomes

The secondary outcomes of this study included perceived stress, depressive symptoms, quality of life, and adherence.

The PSS-10 was administered to measure the stress level of the students. It is a 5-point Likert scale, with scores ranging from 0 to 40 [54].

Depressive symptoms were measured using PHQ-9, which has a 4-point Likert scale. The highest score on PHQ-9 is 27, and high scores represent high levels of depressive symptoms [55].

Quality of life was assessed using the EQ-5D-5L [62]. An index value (EQ-5D-5L index) could be calculated based on 5 dimensions related to the quality of life, namely, mobility, self-care, usual activities, pain or discomfort, and anxiety or depression. Using the EQ-5D-5L Visual Analogue Scale, participants were asked to rate their perceptions about their overall health ranging from 0 (worst health) to 100 (best health).

All scales used in this study are validated tools that yielded high reliability in previous psychometric evaluations [63,64]. In this study, Cronbach α coefficients were calculated to evaluate the reliability of the assessment tools at the pretest and posttest assessments. The results indicated good internal consistency for CSQ-8 (α=.90), SUS-10 (α=.85), PSS-10 (α=.84), and PHQ-9 (α=.79), whereas EQ-5D-5L showed moderate internal consistency (α=.61) at posttest measurements. At the pretest measurements, we found low to moderate Cronbach α for PSS-10 (α=.58), PHQ-9 (α=.70), and EQ-5D-5L (α=.62). Moreover, all questions and scales in this study were provided in both English and Dutch languages, and students could choose their preferred language.

#### Additional Measures

We asked participants to evaluate their perceptions about the therapeutic alliance with the e-coach using the Working Alliance Inventory for Internet Interventions (WAI-I) [65]. WAI-I consists of 12 items, and participants could rate each item on a 5-point Likert scale. High scores indicate high quality of relationship with the e-coach. 

Moreover, at the end of each session, participants rated the usefulness of the session on a scale from 0 (not useful at all) to 100 (very useful). Following this, they shared their evaluations of the specific session, answering several questions developed for another study [66] (Multimedia Appendix 4).

For noncompleters, we sent a questionnaire inquiring about their reasons for not continuing (Multimedia Appendix 5) in addition to posttest assessments. Participants could select multiple reasons for discontinuation.

In addition, during the pretest assessment, participants’ sociodemographic characteristics including age, sex, university, marital status, and current use of formal help were collected. 

#### Semistructured Interviews

We conducted semistructured interviews with both intervention completers and noncompleters. Initially, we aimed to reach students with diverse scores on CSQ-8, SUS-10, and PSS-10. However, owing to a very low response rate, we conducted interviews based on the accessibility and availability of the students. During interviews, a separate question list was used for each group. For the interviews with completers, we used the revised version of the questions that were developed for another study [67]. We also prepared a question list for noncompleters. All questions are available in Multimedia Appendix 6. 

### Data Analysis

We used SPSS (version 25; IBM Corp) for the quantitative analyses. We examined the baseline sociodemographic and clinical characteristics of the whole sample, study completers (ie, participants who completed the posttest assessment) versus noncompleters (ie, participants who did not complete the posttest assessment), and intervention completers (ie, participants who followed at least 3 sessions out of 5) versus noncompleters (ie, participants who completed <3 sessions). Depending on the type of the outcome variable (ie, continuous vs categorical) and the normal distribution indications for the continuous variables, we used the chi-squared test, independent sample *t* tests, or Mann-Whitney *U* test to examine the potential differences between study completers versus noncompleters and intervention completers versus noncompleters at the baseline assessment. 

We calculated the descriptive statistics for our primary outcomes of client satisfaction (CSQ-8) and usability of the intervention (SUS-10) for the whole sample and separately for intervention completers and noncompleters. For the secondary outcomes, we examined whether intervention completers reported statistically significant differences between pretest and posttest scores of perceived stress, depression, and quality of life using paired sample *t* tests (2-tailed) or Wilcoxon signed rank test depending on the indications for normal distribution. We calculated the Cohen *d* effect size, interpreting the benchmarks of 0.2, 0.5, and 0.8 as small, moderate, and large, respectively [68]. Cohen *d* values were obtained by dividing the difference between the mean scores by the SD of the difference scores [69,70].

For qualitative analysis, we first obtained verbatim transcriptions from the audio recordings of the interview. We conducted inductive thematic analysis using the step-by-step approach proposed by Braun and Clarke [71]. Following these steps, we first familiarized the data by reading the transcripts multiple times. Second, we generated the initial codes. Then, 2 assessors (YA and MC) reread the transcriptions and separately identified codes in this procedure. Third, we created tentative themes by merging the codes that possibly have common ground. As a fourth step, the themes were rearranged and revised more meaningfully after discussing with other researchers with expertise in qualitative analysis, internet-based interventions, and mental health of university students (MD, LMdW, and EK). Next, we finalized the definitions of each theme and subtheme.

### Progression Criteria

Progression criteria are useful for assessing the feasibility findings and deciding whether a definitive RCT could be conducted without any changes (green), with changes (amber), or if the trial should be stopped completely in case of concerning situations (red). Limited guidelines exist regarding the interpretation of the feasibility studies’ outcomes. Some studies rely on progression criteria that have been set based on metrics of recruitment, adherence, and outcome data [72]. We determined the progression criteria as follows:

1. At least 70% of the participants had to adhere to the core sessions and fully complete the posttest assessments
2. Completers had to show high satisfaction (overall mean score of CSQ-8 ≥20) and positive user experience (defined by the overall mean score of SUS-10 >80)
3. Participants who completed the intervention had to show significant improvement in stress scores assessed using PSS-10 from baseline to posttest assessment.

## Results

### Participants

A total of 1004 students registered for the intervention from June 2020 until March 14, 2022. Of the 1004 registered students, 436 (43.43%) met the eligibility criteria. The reasons for exclusion were low scores on PSS-10 or high scores on PHQ-9 (229/1004, 22.81%), incomplete baseline data (202/1004, 20.12%), not giving informed consent (65/1004, 6.47%), and still following the intervention or being moved to another intervention in the Caring Universities project after discussing this with the e-coach and supervision team (71/1004, 7.07%).

The average age of the whole sample was 22.89 (SD 0.17) years, and most participants were female (382/436, 87.6%). More than half of the students were from the Netherlands (272/436, 62.4%), whereas other students were from various other countries, mainly from Europe (136/436, 31.2%). Approximately half of the participants (203/436, 46.6%) were master’s degree students, and the remaining were primarily undergraduate students (223/436, 51.1%). Most students (367/436, 84.2%) reported not currently receiving professional help. Further details are provided in Multimedia Appendix 7.

Among the students who met the eligibility criteria, 29.6% (129/436) did not start using the intervention despite showing interest. Approximately half of the noninitiators did not activate their account (62/129, 48.1%), whereas others activated their account but did not open the intervention page (50/129, 38.8%) or quit the intervention before completing the introduction session (17/129, 13.2%). Table 1 shows the details about the uptake and completion rates for each session.

**Figure 1:**
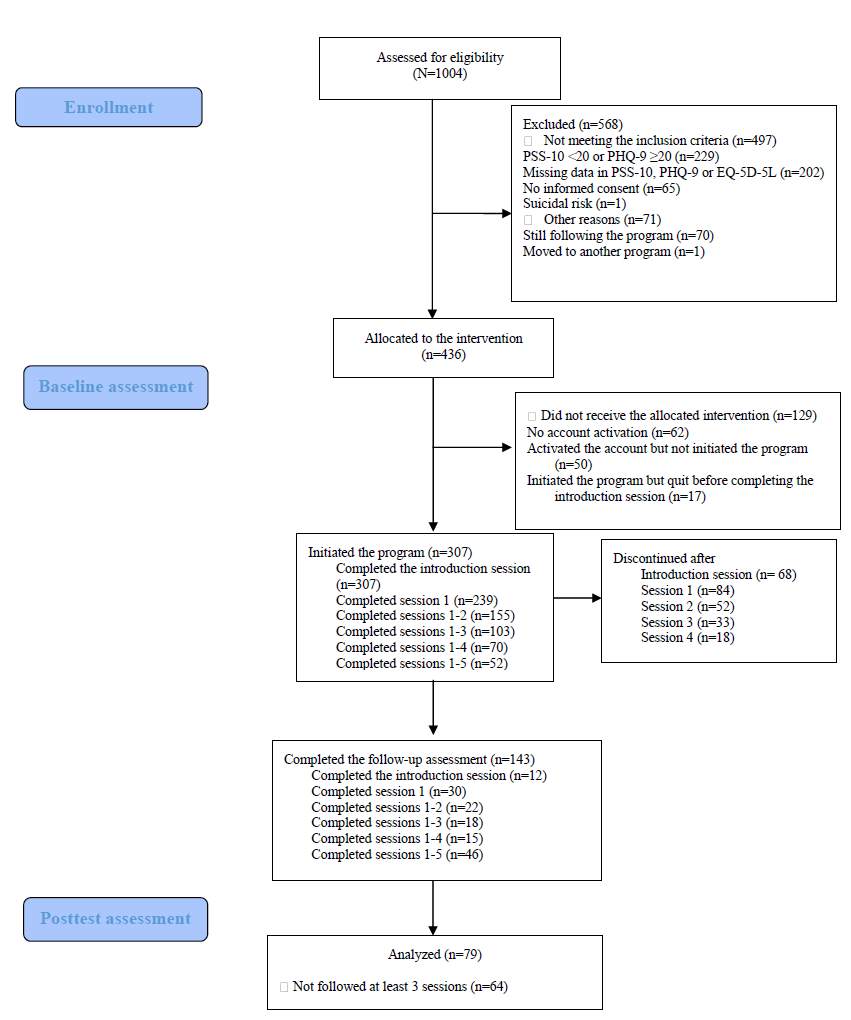
CONSORT (Consolidated Standards of Reporting Trials) 2010 flow diagram. PHQ-9: Patient Health Questionnaire–9; PSS-10: Perceived Stress Scale–10.

Of all students who initiated the intervention, more than half (204/307, 66.4%) discontinued within the first 2 sessions (84/204, 41.2% stopped after the first session). In total, 103 students were intervention completers. The intervention completion rate was 33.6% (103/307). However, not all participants who completed the intervention provided the posttest assessment. Of the 307 participants who initiated the intervention, 79 (25.7%) were intervention completers. Only a small number of students followed the optional sessions. Among the 4 optional sessions, the most commonly used session was about time management and procrastination (42/307, 13.7%). Following this, 10.7% (33/307) of students completed the optional session about healthy lifestyles. The sessions about assertiveness and adaptation to a new culture session were used by 7.5% (23/307) and 2.9% (9/307) of students, respectively. The flowchart and detailed information regarding the number of students who completed each session can be seen in Table 1 and Figure 1.

**Table 1:** Uptake of the intervention and completion rates of each session.

			Participants, n (%)
**Uptake of the intervention (N=436)^a^**			
	Account activated	374 (85.8)	
	Intervention initiated	324 (74.3)	
	Completed the introduction session	307 (70.4)	
	Completed session 1	239 (54.8)	
	Completed sessions 1-2	155 (35.6)	
	Completed sessions 1-3	103 (23.6)	
	Completed sessions 1-4	70 (16.1)	
	Completed sessions 1-5	52 (11.9)	
**Completed the optional sessions (n=307)^b^**			
	Assertiveness	23 (7.5)	
	Adaptation to a new culture	9 (2.9)	
	Time management and procrastination	42 (13.7)	
	Sleeping, eating, and exercising	33 (10.7)	

^a^Percentages were calculated based on the 436 participants who showed interest in participating.

^b^Percentages were calculated based on the 70.4% (307/436) of participants who initiated the intervention.

We found no significant differences between intervention completers and noncompleters in terms of baseline sociodemographic and clinical characteristics except the enrolled university and depression level. Our results suggested that intervention noncompleters reported higher scores on PHQ-9 (mean 10.67, SD 4.26) than completers (mean 9.48, SD 3.80; *P*=.01; Multimedia Appendix 7).

### Primary Outcomes

The average score of students who completed the intervention on CSQ-8 was 25.78 (SD 3.30; range 16-31), indicating good satisfaction [45]. The mean score for SUS-10 was 86.01 (SD 10.25; range 50-100), indicating a very good or excellent user experience [61,73]. Noncompleters reported significantly low satisfaction (mean 22.70, SD 3.77) compared with completers (mean 25.78, SD 3.30; *P*<.001; Cohen *d*=−0.87). Moreover, noncompleters reported lower usability scores (mean 80.74, SD 13.92) than completers (mean 86.01, SD 10.25; *P*=.01; Cohen *d*=−0.50).

The adherence rate for the intervention was 33.6% (103/307). Given that not all intervention completers provided data at the posttest assessment, only 25.7% (79/307) of the students fully engaged in the study protocol by completing the core sessions and providing complete data at posttest assessment. On average, participants completed 2.01 (SD 1.74) sessions, representing 40% of the whole intervention.

### Secondary Outcomes

Participants who completed the intervention showed significant improvements in perceived stress from baseline (mean 25.16, SD 3.70) to posttest assessment (mean 20.66, SD 5.77; *t*_78_=7.15; *P*<.001), indicating a large effect size (Cohen *d=*0.80). Depression scores also significantly decreased from baseline (mean 9.46, SD 3.77) to posttest assessment (mean 7.52, SD 4.33; *t*_78_=4.18; *P*<.001), yielding a moderate effect size (Cohen *d=*0.47). Results also showed a moderate effect size for quality of life (Cohen *d=*−0.35)*,* from baseline (mean 0.72, SD 0.12) to posttest assessment (mean 0.77, SD 0.15; *z* score=−3.18; *P*=.001; Table 2).

**Table 2:** Changes from baseline to posttest main analysis (n=79).

Secondary outcomes	Baseline assessment score, mean (SD)	Posttest assessment score, mean (SD)	*t* test *(df)*	*z* score	*P* value	Cohen *d*
PSS-10^a,b^	25.16 (3.70)	20.66 (5.77)	7.15 (78)	N/A^c^	<.001	0.80
PHQ-9^b,d^	9.46 (3.77)	7.52 (4.33)	4.18 (78)	N/A	<.001	0.47
EQ-5D-5L-index^e^	0.72 (0.12)	0.77 (0.15)	N/A	−3.18	.001	−0.35
EQ-5D-5L-VAS^b^	57.41 (18.94)	72.18 (15.14)	−6.60 (78)	N/A	<.001	−0.74

^a^PSS-10: Perceived Stress Scale–10.

^b^2-tailed paired sample *t* test.

^c^N/A: not applicable; 2-tailed paired sample *t* tests were used for the comparisons.

^d^PHQ-9: Patient Health Questionnaire–9.

^e^Wilcoxon signed rank test.

### Additional Measures

Among the 79 intervention completers, 75 (95%) participants provided complete data on WAI-I (Table S1 in Multimedia Appendix 8). The average score for WAI-I was 46.37 (SD 6.73), and this is comparable with the results of other guided, internet-based, or face-to-face interventions [65,74-76].

For the 5 main sessions, participants evaluated the usefulness of the intervention positively, with average scores ranging from 75 to 83. The lowest score was for the psychoeducation of stress (mean 74.93, SD 17.76), and the highest score was for the cognitive restructuring session (mean 82.83, SD 12.20; Tables S2 and S3 in Multimedia Appendix 8). Students reported moderate usefulness for the optional sessions (Tables S2 and S4 in Multimedia Appendix 8).

Among all the 333 intervention noncompleters, 42 (12.6%) students replied to the survey inquiring about the reasons for discontinuation. The most common reasons were loss of interest or motivation (23/42, 55%), lack of time (18/42, 43%), preference for another source of help (13/42, 31%), not being able to find the needed information in the intervention (9/42, 21%), and perceiving the intervention as very demanding (6/42, 14%; Table S5 in Multimedia Appendix 8).

### Results of the Semistructured Interviews

#### Overview

We conducted semistructured interviews with 11 participants who completed at least 3 sessions of the intervention and 7 participants who started using the intervention but discontinued the intervention within the first 3 sessions. Each interview lasted approximately 30 minutes. As a result of the qualitative analysis, we generated four overarching themes that are associated with the feasibility and acceptability of the intervention: (1) considerations of initiating the intervention, (2) intervention-related factors influencing user experience, (3) appraisals of experienced support by the e-coach, and (4) personal factors interfering with the user experience. We also generated a theme representing the users’ suggestions for improvement of the intervention. Here, we provide the key highlights of each theme, and extended explanations are available in Table S1 in Multimedia Appendix 9.

#### Considerations of Initiating the Intervention

This theme describes the preintervention experiences of the students initiating the intervention. Students highlighted the importance of the heightened perceived need for the intervention. The timing of the intervention was influential for initiation as several students reported that they started the intervention particularly when they were experiencing a stressful period. A participant reported the following:

I do think the program came at a very good time for me because at the moment, at that time, I was very stressed, it was the end of the year. So there were a lot of things to do with exams and my work and my job. So a lot of things came together and had to be arranged and done. So at that time, I was very stressed out and it was very helpful to arrange some things for myself, and the modules really helped with that.

Completer 1

External factors such as referral from the student psychologist or screening results facilitated the uptake of the intervention for some students. Some started the intervention without clear expectations. The accessibility and flexibility of the intervention and the presence of guidance were also identified as important factors influencing their decision to start the intervention.

#### Intervention-Related Factors Influencing User Experience

Students expressed a variety of elements related to the intervention and its features that affected their experience. The attractiveness of the user interface, usability of the intervention and its features, relevance of the content, convenience of the tailoring and personalization, and helpfulness of the intervention were identified as the prominent factors affecting their overall experience.

In general, students found the intervention to be visually attractive. Many evaluated the intervention as easy to use and not very demanding. In contrast, some students, particularly noncompleters, considered the intervention to be challenging because of the time and effort required to complete it. Most students found the intervention to be relevant and helpful. Some students shared the positive effects of the intervention, such as increasing awareness, improving their stress symptoms, and obtaining more positive attitudes toward seeking formal help or taking action to see a mental health professional. However, some students mentioned a mismatch between their needs and the intervention’s content. For instance, some students reported that they needed more concrete stress management techniques, whereas others reported their changing objectives for dealing with low mood instead of high stress. A few students commented about the lack of novelty or practical activities as the negative aspect of the intervention.

Generally, students appreciated the flexibility of the intervention and its adjustable features such as the availability of optional sessions. However, some students mentioned the need for more advanced personalization components, particularly regarding the adjustable length and content of the intervention and the feedback provided by the e-coach. Perceived lack of interaction was another criticism, as some students underscored the need for more communication (eg, face-to-face interaction or more frequent or synchronous conversations with the e-coach).

#### Appraisals of Experienced Support From the e-Coach

This theme represents the students’ experiences with the received support. The central focus of this theme is to what extent and in what way they felt supported by the e-coach. Students reported emotional support and informational support. Many valued the e-coach’s feedback and encouragement, but a few students expressed skepticism toward the professionalism of the e-coach. For example, a participant reported her concerns as follows:

I know she [e-coach] would understand this, but maybe her expertise that I feel like...not for me or something. So maybe that also plays a role.... I think it’s pretty good but like she felt a little bit too young and a little bit too less experienced about my problems I’m having.

Dropout 4

#### Personal Factors Interfering With the User Experience

This theme includes the personal aspects that might affect intervention experiences. Students mentioned that having a busy schedule and lack of motivation were barriers to intervention use. Some students also reported discontinuation because of the lack of perceived need for a stress-related intervention, whereas a few reported the need for more personalized or extended help such as individual psychotherapy.

#### Suggestions for Improvement of the Intervention

This theme was generated based on the students’ recommendations for optimizing the intervention and enhancing the user experience. The recommendations were made based on four aspects: (1) enhancing the attractiveness of the user interface and improving usability, (2) optimizing the intervention content, (3) improving support and interaction, and (4) improving the implementation and integration of the intervention. Although most participants reported high satisfaction with the user interface, a few students suggested the option for selecting different layouts, such as having a more minimalistic outlook. Students shared their suggestions for enriching the content by adding more practical activities with increased variety in terms of length and optional sessions. Some students suggested increasing social interaction, for instance, by adding a forum page, increasing communication with the e-coach, and organizing face-to-face meetings with the e-coach. Finally, students had recommendations for the implementation of the intervention, such as an integration of the internet interventions into a broad context within the higher education setting where other student services are interconnected.

## Discussion

### Principal Findings

This study aimed to investigate the feasibility, acceptability, and adherence of a new internet-based stress management intervention supported by an e-coach for university students with high levels of stress. The students reported high satisfaction and usability of the intervention. Students who completed the intervention showed medium to large positive effects for perceived stress, depression, and quality of life from baseline to posttest assessment. However, low adherence rates indicate possible challenges, which might jeopardize the maximal use of the intervention. On the basis of our progression criteria and qualitative findings, our feasibility trial was deemed to be amber, meaning that some modifications in the protocol are required, especially to increase adherence rates before progressing to the definitive RCT. Findings from the qualitative analyses provided some important insights about how to improve the impact of the intervention targeting factors at the individual (eg, increasing motivation and using facilitators for uptake and use), intervention (eg, improving flexibility and personalization, increasing interaction, and integrating mental health professionals when needed), and organizational levels (eg, integrating student services and promoting the intervention).

### Comparison With Previous Studies

Similar to our results on satisfaction, other studies reported that university students with different mental health problems reported high satisfaction levels with internet-based interventions [77,78]. Our finding regarding intervention usability also was comparable with those of similar interventions for stress in university students [79,80]. As expected, the internet-based intervention for stress management appeared to be suitable for university students. In addition to scores on satisfaction and usability, the dropout rate could be a marker of the acceptability of the intervention. Worldwide, internet-based interventions for psychological disorders yielded dropout rates between 2% and 83%, with a mean score of 31% [81]. Although the dropout rate of 74.3% (228/307) in our study aligns with this range, it is still high when compared with other internet-based stress management interventions focusing primarily on university students with high levels of stress [29,82,83] and digital mental health interventions for children and adolescents [84] in which dropout rates were found to be up to 30%.

A myriad of factors, including personal variables, intervention or technology features, and contextual circumstances, can play roles in low adherence [31,85-87]. In addition, reasons for discontinuation may differ according to the time point of the dropout [88]. For instance, in our study, 25.7% (112/436) of the students did not start the intervention after registration. This refers to preintervention dropout, commonly observed in psychological interventions, particularly in eHealth interventions [28,81,89]. Theoretical models explaining health behaviors and previous studies of adherence suggest that low motivation, feeling not ready to change, lack of perceived need, or perceived costs of following the intervention (eg, feelings of discomfort during behavior change and required time and effort) could be barriers to initiating the intervention [85,90-93]. In this study, the reasons for preintervention dropout remained unclear.

Provision of incentives, such as financial compensation or course credit, is widely used to stimulate intervention or study adherence. Although some studies showed a positive effect of such external rewards on the likelihood of increasing engagement, systematic reviews revealed mixed findings [26,94,95]. For example, a study found that commitment to the intervention may not be associated with financial compensation [95]. Another meta-analysis of digital interventions for university students found a significant association between the absence of compensation and high effect size [26]. Therefore, the use of incentives is a contentious issue and, possibly, is not the silver bullet that can prevent dropout.

Among the students who started the intervention, discontinuation usually occurred during the first 2 sessions, meaning that many students were not exposed to the core part of the intervention. Our qualitative data and dropout survey demonstrated that the interplay between personal and intervention characteristics plays a key role in dropout at this stage. Regarding personal reasons, having a busy schedule was reported as a top barrier. Although internet-based interventions are renowned for their flexibility and easy access regarding time and place, time constraints still appear to be a challenge to keeping participants in the intervention [85,93]. Students in higher education might not prioritize intervention use among heavy workloads, increased study or life responsibilities, and external demands (eg, submitting an assignment on time). This also raises questions about whether single-session or one-at-a-time therapy approaches could be an alternative way for some students. Although there is recent evidence accentuating the possibility of compressing multiple-session interventions into single-session interventions, more studies should be conducted to gain more knowledge about this topic [96,97].

Change in symptom severity, referring to the continuum from improvement in the negative state to worsening symptoms, could be another factor that influences adherence [98]. Some students might quit the intervention at an early stage because they might promptly gain some benefits, as findings showed that even brief psychoeducation about stress might result in positive effects for some participants [99,100]. Alternatively, the underlying reason for dropout for some students can be worsening symptoms because of the intervention itself or general symptomology changes [31,101]. Face-to-face or blended interventions could fit better with their augmented symptoms than an internet-based intervention, as we know that some of them started individual therapy.

Although some students reported the benefits of existing personalization components of our intervention, we identified that students generally preferred more advanced personalization elements. Offering adjustable content or features based on user preferences is essential in designing eHealth interventions [102]. The provision of such flexibility could align with the developmental characteristics of young populations, as it reinforces autonomy [103]. In this study, several students commented about the importance of increasing the diversity of optional modules and case examples, providing more extended communication with the e-coach, and offering stress management exercises of varying lengths as alternative ways to increase personalization.

Some students also felt the need for more interaction not only with e-coach but also with other users by integrating forums into the intervention, where students can disclose themselves and exchange ideas. Various theoretical models, such as Persuasive System Design [102], Behavior Change Model for Internet Interventions [87], and Behavioral Intervention Technology Model [104], asserted that social support components in eHealth interventions may increase adherence to and impact of the intervention. Although interactive components could evoke a sense of relatedness, which is a fundamental psychological need [105,106], and students might feel more supported or understood, the effects of such supplementary components remain unclear [107-109].

Another important factor affecting the user experience was the content of the intervention. We found that most completers evaluated the content as relevant and helpful. Relevance of the information was found as an important factor in adherence to and user experience with eHealth interventions [31]. However, some students, particularly noncompleters, addressed the demandingness of the intervention in terms of exercises and length, insufficient number of practical activities, and lack of novelty of the content as drawbacks. Some students (eg, psychology students) might be already familiar with the content, and this might have evoked feelings of, for example, boredom. This, once more, brings the importance of personalization to the agenda, which is a critical element in meeting the heterogonous needs of university students.

Although this project was not designed for the pandemic circumstances, the COVID-19 pandemic occurred during the implementation. Therefore, while interpreting these findings, we should not neglect the contextual factors that might affect the user experience. As we captured in the interviews, some students voiced that they would have preferred to seek help from in-person resources instead of an internet-based intervention if there had been no pandemic restrictions. Similarly, conducting almost all educational and extracurricular activities in the web-based environment possibly influenced adherence. For example, studies have pointed out that students’ stress levels increased during the pandemic, which was found to be associated with difficulties in balancing study and life [110,111]. Among these factors, students might be overwhelmed by online activities, which can cause screen fatigue and reluctance to follow internet-based interventions.

In general, we observed low levels of engagement with the optional sessions, although qualitative findings showed that students wished for more optional sessions. Adaptation to a new culture had the lowest completion rates by far. This session could be very specific to international students and our sample predominantly consisted of domestic students. Another explanation could be that international students could have already acquired this knowledge from other events organized by the university. In the main sessions, students generally did not follow sessions 4 and 5, possibly because they lost interest toward the end of the intervention or they had already gained specific skills in the core sessions.

By blending the results from other studies with our findings, some recommendations can be provided for optimizing our intervention. Students with high stress might benefit from the internet-based stress management intervention; however, keeping them in the intervention is challenging. Therefore, a priori strategies should be planned and implemented to sustain successful engagement. Extended personalization should be provided, as participants might have assorted preferences, needs, and backgrounds, consistent with other studies [112-114]. This could be achieved by improving the diversity of optional modules, which cover the topics of different stress sources, and integrating enriched opportunities such as adjustable content and activities. To increase adherence, sending notifications as a reminder seemed to be an effective approach; however, as some students reported, email reminders could be easily missed. Push notifications (eg, user’s selection of preferred time, modality, or frequency) could be studied further to find the most effective method.

A strategy could be to alter the modality and frequency of communication with the e-coach. Considering the required resources for providing extended communication, the impact of other types of guidance, for example, guidance on demand, should be investigated. Future studies should tackle the impact of on-demand guidance, given the evidence of its effectiveness in previous studies [115,116]. As some students reported their dropout reason as the absence of mental health professionals, building a stepped-care framework where students can communicate with a mental health professional when needed could be a good alternative [117]. In addition, an online forum where users can share their ideas about stress and its management should be tested.

### Limitations

Our study is not free from limitations. First, the dropout rate was high, potentially threatening the internal and external validity of the study. Therefore, our results require careful interpretation. Moreover, the vague dropout reasons of students who showed interest but did not start the intervention limit our knowledge about intervention use. Both quantitative and qualitative data should be collected to understand preintervention dropout reasons during the future application of our intervention. Second, the sample mainly consisted of female students. Therefore, the understanding of the experiences of other genders is limited. Future studies should include a broad representation of gender categories, encompassing the entire gender spectrum. Third, although it was beyond the scope of this study, it is important to acknowledge that the single-arm study design provides us with restricted information about the intervention effects. The observed improvements could be attributed to various random factors or the regression to the mean. Consequently, the interpretation of the symptom change captured from baseline to posttest assessment should be interpreted with caution. As a next step, after optimizing the intervention, we plan to move forward by conducting an RCT in which we test the effects of the intervention in comparison with the control group and perform analysis with the intent-to-treat principle to mitigate potential bias arising from dropout. Fourth, we did not set an exclusion criterion regarding the maximum age of students; however, we acknowledge that stress experiences could differ in older versus younger students. Such potential difference could be reflected in the intervention in a more salient way, for example, by enriching the intervention with examples or optional sessions that address the unique problems of different age groups.

### Conclusions

In conclusion, the internet-based stress management intervention seems to be feasible, acceptable, and plausibly effective, especially for those who had time and intrinsic motivation to follow the intervention. The high dropout rate signals a need for intervention amendments to increase adherence. Several suggestions have been made to improve the intervention, including increasing human support, incorporating a mental health professional, and integrating more practical exercises into the platform. Particularly, qualitative data obtained from lived experiences could provide a rich seam of information to understand the variety of user experiences fully. This can help us determine prudent and user-centric strategies for efficient intervention delivery.

## Appendix

Multimedia Appendix 1 Changes to the protocol.

Multimedia Appendix 2 CONSORT-EHEALTH (Consolidated Standards of Reporting Trials of Electronic and Mobile Health Applications and Online Telehealth; version 1.6.1) checklist.

Multimedia Appendix 3 Screenshots of the intervention.

Multimedia Appendix 4 Questions regarding session evaluation.

Multimedia Appendix 5 Survey questions for noncompleters.

Multimedia Appendix 6 Questions for semistructured interviews.

Multimedia Appendix 7 Baseline characteristics of the participants.

Multimedia Appendix 8 Tables of additional outcomes.

Multimedia Appendix 9 Extended results of the semistructured interviews.

## Abbreviations

CBT: cognitive behavioral therapy
CONSORT-EHEALTH: Consolidated Standards of Reporting Trials of Electronic and Mobile Health Applications and Online Telehealth
CSQ-8: Client Satisfaction Questionnaire–8
PHQ-9: Patient Health Questionnaire–9
PSS-10: Perceived Stress Scale–10
RCT: randomized controlled trial
SUS-10: System Usability Scale–10
WAI-I: Working Alliance Inventory for Internet Interventions
WMH-ICS: World Mental Health International College Student Initiative

## Data Availability

The data sets generated during and/or analyzed during this study are available from the corresponding author on reasonable request

## References

[1] de Girolamo G, Dagani J, Purcell R, Cocchi A, McGorry PD (2012). Age of onset of mental disorders and use of mental health services: needs, opportunities and obstacles. Epidemiol Psychiatr Sci.

[2] Gorman KS, Bruns C, Chin C, Fitzpatrick NY, Koenig L, LeViness P, Sokolowski K (2021). Annual survey: 2020. Association for University and College Counseling Center Directors.

[3] Hartson KR, Hall LA, Choate SA (2023). Stressors and resilience are associated with well-being in young adult college students. J Am Coll Health.

[4] Pedrelli P, Nyer M, Yeung A, Zulauf C, Wilens T (2015). College students: mental health problems and treatment considerations. Acad Psychiatry.

[5] (2019). American College Health Association: National College Health Assessment II: reference group executive summary spring 2019. American College Health Association.

[6] Pierceall EA, Keim MC (2007). Stress and coping strategies among community college students. Community Coll J Res Pract.

[7] Stoliker BE, Lafreniere KD (2015). The influence of perceived stress, loneliness, and learning burnout on university students' educational experience. Coll Stud J.

[8] Karyotaki E, Cuijpers P, Albor Y, Alonso J, Auerbach RP, Bantjes J, Bruffaerts R, Ebert DD, Hasking P, Kiekens G, Lee S, McLafferty M, Mak A, Mortier P, Sampson NA, Stein DJ, Vilagut G, Kessler RC (2020). Sources of stress and their associations with mental disorders among college students: results of the World Health Organization world mental health surveys international college student initiative. Front Psychol.

[9] Lund HG, Reider BD, Whiting AB, Prichard JR (2010). Sleep patterns and predictors of disturbed sleep in a large population of college students. J Adolesc Health.

[10] Tudehope L, Lee P, Wiseman N, Dwirahmadi F, Sofija E (2022). The effect of resilience on the relationship between perceived stress and change in alcohol consumption during the COVID-19 pandemic in Queensland, Australia. J Health Psychol.

[11] Gulewitsch MD, Enck P, Schwille-Kiuntke J, Weimer K, Schlarb AA (2013). Mental strain and chronic stress among university students with symptoms of irritable bowel syndrome. Gastroenterol Res Pract.

[12] Cohen S, Janicki-Deverts D, Miller GE (2007). Psychological stress and disease. JAMA.

[13] Eisenberg D, Lipson SK, Ceglarek P, Kern A, Phillips MV, Cimini MD, Rivero EM (2018). College student mental health: the national landscape. Promoting Behavioral Health and Reducing Risk among College Students.

[14] Le LK, Esturas AC, Mihalopoulos C, Chiotelis O, Bucholc J, Chatterton ML, Engel L (2021). Cost-effectiveness evidence of mental health prevention and promotion interventions: a systematic review of economic evaluations. PLoS Med.

[15] Kuster AT, Dalsbø TK, Luong Thanh BY, Agarwal A, Durand-Moreau QV, Kirkehei I (2017). Computer-based versus in-person interventions for preventing and reducing stress in workers. Cochrane Database Syst Rev.

[16] Ong L, Linden W, Young S (2004). Stress management: what is it?. J Psychosom Res.

[17] Amanvermez Y, Rahmadiana M, Karyotaki E, de Wit L, Ebert DD, Kessler RC, Cuijpers P (2020). Stress management interventions for college students: a systematic review and meta-analysis. Clin Psychol Sci Pract.

[18] Regehr C, Glancy D, Pitts A (2013). Interventions to reduce stress in university students: a review and meta-analysis. J Affect Disord.

[19] Yusufov M, Nicoloro-SantaBarbara J, Grey NE, Moyer A, Lobel M (2019). Meta-analytic evaluation of stress reduction interventions for undergraduate and graduate students. Int J Stress Manag.

[20] Czyz EK, Horwitz AG, Eisenberg D, Kramer A, King CA (2013). Self-reported barriers to professional help seeking among college students at elevated risk for suicide. J Am Coll Health.

[21] Ebert DD, Mortier P, Kaehlke F, Bruffaerts R, Baumeister H, Auerbach RP, Alonso J, Vilagut G, Martínez KI, Lochner C, Cuijpers P, Kuechler AM, Green J, Hasking P, Lapsley C, Sampson NA, Kessler RC (2019). Barriers of mental health treatment utilization among first-year college students: first cross-national results from the WHO World Mental Health International College Student Initiative. Int J Methods Psychiatr Res.

[22] Ebert DD, Van Daele T, Nordgreen T, Karekla M, Compare A, Zarbo C, Brugnera A, Øverland S, Trebbi G, Jensen KL, Kaehlke F, Baumeister H (2018). Internet- and mobile-based psychological interventions: applications, efficacy, and potential for improving mental health. Eur Psychol.

[23] Murray E, Hekler EB, Andersson G, Collins LM, Doherty A, Hollis C, Rivera DE, West R, Wyatt JC (2016). Evaluating digital health interventions: key questions and approaches. Am J Prev Med.

[24] Eustis EH, Hayes-Skelton SA, Orsillo SM, Roemer L (2018). Surviving and thriving during stress: a randomized clinical trial comparing a brief web-based therapist-assisted acceptance-based behavioral intervention versus waitlist control for college students. Behav Ther.

[25] Dunbar MS, Sontag-Padilla L, Kase CA, Seelam R, Stein BD (2018). Unmet mental health treatment need and attitudes toward online mental health services among community college students. Psychiatr Serv.

[26] Harrer M, Adam SH, Baumeister H, Cuijpers P, Karyotaki E, Auerbach RP, Kessler RC, Bruffaerts R, Berking M, Ebert DD (2019). Internet interventions for mental health in university students: a systematic review and meta-analysis. Int J Methods Psychiatr Res.

[27] Davies EB, Morriss R, Glazebrook C (2014). Computer-delivered and web-based interventions to improve depression, anxiety, and psychological well-being of university students: a systematic review and meta-analysis. J Med Internet Res.

[28] Wangberg SC, Bergmo TS, Johnsen JA (2008). Adherence in Internet-based interventions. Patient Prefer Adherence.

[29] Hintz S, Frazier PA, Meredith L (2015). Evaluating an online stress management intervention for college students. J Couns Psychol.

[30] Wojtowicz M, Day V, McGrath PJ (2013). Predictors of participant retention in a guided online self-help program for university students: prospective cohort study. J Med Internet Res.

[31] Borghouts J, Eikey E, Mark G, De Leon C, Schueller SM, Schneider M, Stadnick N, Zheng K, Mukamel D, Sorkin DH (2021). Barriers to and facilitators of user engagement with digital mental health interventions: systematic review. J Med Internet Res.

[32] Fleischmann R, Harrer M, Zarski A, Baumeister H, Lehr D, Ebert D (2017). Patients' experiences in a guided internet- and app-based stress intervention for college students: a qualitative study. Internet Interv.

[33] Kvillemo P, Brandberg Y, Bränström R (2016). Feasibility and outcomes of an internet-based mindfulness training program: a pilot randomized controlled trial. JMIR Ment Health.

[34] Amanvermez Y, Zhao R, Cuijpers P, de Wit LM, Ebert DD, Kessler RC, Bruffaerts R, Karyotaki E (2022). Effects of self-guided stress management interventions in college students: a systematic review and meta-analysis. Internet Interv.

[35] Conley CS, Durlak JA, Shapiro JB, Kirsch AC, Zahniser E (2016). A meta-analysis of the impact of universal and indicated preventive technology-delivered interventions for higher education students. Prev Sci.

[36] Conley CS, Shapiro JB, Kirsch AC, Durlak JA (2017). A meta-analysis of indicated mental health prevention programs for at-risk higher education students. J Couns Psychol.

[37] Palacios JE, Richards D, Palmer R, Coudray C, Hofmann SG, Palmieri PA, Frazier P (2018). Supported internet-delivered cognitive behavioral therapy programs for depression, anxiety, and stress in university students: open, non-randomised trial of acceptability, effectiveness, and satisfaction. JMIR Ment Health.

[38] Bowen DJ, Kreuter M, Spring B, Cofta-Woerpel L, Linnan L, Weiner D, Bakken S, Kaplan CP, Squiers L, Fabrizio C, Fernandez M (2009). How we design feasibility studies. Am J Prev Med.

[39] Orsmond GI, Cohn ES (2015). The distinctive features of a feasibility study: objectives and guiding questions. OTJR (Thorofare N J).

[40] LeBlanc J, Talbot F, Fournier V, Titov N, Dear BF (2022). Lessons learned from two feasibility trials of a translated and minimally monitored iCBT program for young adults among community and university samples. Internet Interv.

[41] Mol M, van Schaik A, Dozeman E, Ruwaard J, Vis C, Ebert DD, Etzelmueller A, Mathiasen K, Moles B, Mora T, Pedersen CD, Skjøth MM, Pensado LP, Piera-Jimenez J, Gokcay D, Ince BÜ, Russi A, Sacco Y, Zanalda E, Zabala AF, Riper H, Smit JH (2020). Dimensionality of the system usability scale among professionals using internet-based interventions for depression: a confirmatory factor analysis. BMC Psychiatry.

[42] Sieverink F, Kelders SM, van Gemert-Pijnen JE (2017). Clarifying the concept of adherence to eHealth technology: systematic review on when usage becomes adherence. J Med Internet Res.

[43] Donkin L, Christensen H, Naismith SL, Neal B, Hickie IB, Glozier N (2011). A systematic review of the impact of adherence on the effectiveness of e-therapies. J Med Internet Res.

[44] Hornbæk K, Hertzum M (2017). Technology acceptance and user experience: a review of the experiential component in HCI. ACM Trans Comput Hum Interact.

[45] Boß L, Lehr D, Reis D, Vis C, Riper H, Berking M, Ebert DD (2016). Reliability and validity of assessing user satisfaction with web-based health interventions. J Med Internet Res.

[46] Lattie EG, Adkins EC, Winquist N, Stiles-Shields C, Wafford QE, Graham AK (2019). Digital mental health interventions for depression, anxiety, and enhancement of psychological well-being among college students: systematic review. J Med Internet Res.

[47] Garrido S, Millington C, Cheers D, Boydell K, Schubert E, Meade T, Nguyen QV (2019). What works and what doesn't work? A systematic review of digital mental health interventions for depression and anxiety in young people. Front Psychiatry.

[48] Kaltenthaler E, Sutcliffe P, Parry G, Beverley C, Rees A, Ferriter M (2008). The acceptability to patients of computerized cognitive behaviour therapy for depression: a systematic review. Psychol Med.

[49] Yardley L, Spring BJ, Riper H, Morrison LG, Crane DH, Curtis K, Merchant GC, Naughton F, Blandford A (2016). Understanding and promoting effective engagement with digital behavior change interventions. Am J Prev Med.

[50] Cuijpers P, Auerbach RP, Benjet C, Bruffaerts R, Ebert D, Karyotaki E, Kessler RC (2019). The World Health Organization World Mental Health International College student initiative: an overview. Int J Methods Psychiatr Res.

[51] Amanvermez Y, Karyotaki E, Cuijpers P, Salemink E, Spinhoven P, Struijs S, de Wit LM (2021). Feasibility and acceptability of a guided internet-based stress management intervention for university students with high levels of stress: protocol for an open trial. Internet Interv.

[52] Eysenbach G, CONSORT-EHEALTH Group (2011). CONSORT-EHEALTH: improving and standardizing evaluation reports of Web-based and mobile health interventions. J Med Internet Res.

[53] Cohen S, Kamarck T, Mermelstein R (1983). A global measure of perceived stress. J Health Soc Behav.

[54] Cohen S, Williamson GM, Oskamp S, Spacapan S (1988). Perceived stress in a probability sample of the United States. The Social Psychology of Health: The Claremont Symposium on Applied Social Psychology.

[55] Kroenke K, Spitzer RL, Williams JB (2001). The PHQ-9: validity of a brief depression severity measure. J Gen Intern Med.

[56] Lazarus RS, Folkman S (1984). Stress, Appraisal, and Coping.

[57] Beck AT, Dozois DJ (2011). Cognitive therapy: current status and future directions. Annu Rev Med.

[58] Cuijpers P, Noma H, Karyotaki E, Cipriani A, Furukawa TA (2019). Effectiveness and acceptability of cognitive behavior therapy delivery formats in adults with depression: a network meta-analysis. JAMA Psychiatry.

[59] Larsen DL, Attkisson C, Hargreaves WA, Nguyen TD (1979). Assessment of client/patient satisfaction: development of a general scale. Eval Program Plann.

[60] Brooke J (1996). SUS: A 'quick and dirty' usability scale. Usability Evaluation in Industry.

[61] Lewis JR, Sauro J (2018). Item benchmarks for the System Ssability Scale. J Usability Stud.

[62] (2019). EQ-5D-5L user guide. EuroQol Research Foundation.

[63] Golicki D, Zawodnik S, Janssen MF, Kiljan A, Hermanowski T (2010). Psychometric comparison of EQ-5D and EQ-5D-5L in student population. Value Health.

[64] Lee EH (2012). Review of the psychometric evidence of the perceived stress scale. Asian Nurs Res (Korean Soc Nurs Sci).

[65] Gómez Penedo JM, Berger T, Grosse Holtforth M, Krieger T, Schröder J, Hohagen F, Meyer B, Moritz S, Klein JP (2020). The working alliance inventory for guided internet interventions (WAI-I). J Clin Psychol.

[66] Rahmadiana M, Karyotaki E, Passchier J, Cuijpers P, van Ballegooijen W, Wimbarti S, Riper H (2019). Guided internet-based transdiagnostic intervention for Indonesian university students with symptoms of anxiety and depression: a pilot study protocol. Internet Interv.

[67] Devi R, Carpenter C, Powell J, Singh S (2014). Exploring the experience of using a web-based cardiac rehabilitation programme in a primary care angina population: a qualitative study. Int J Ther Rehabil.

[68] Cohen J (1988). Statistical Power Analysis for the Behavioral Sciences.

[69] Lakens D (2013). Calculating and reporting effect sizes to facilitate cumulative science: a practical primer for t-tests and ANOVAs. Front Psychol.

[70] Neath I Effect size calculator. Psychology Department at Virginia Tech.

[71] Braun V, Clarke V (2006). Using thematic analysis in psychology. Qual Res Psychol.

[72] Avery KN, Williamson PR, Gamble C, O'Connell Francischetto E, Metcalfe C, Davidson P, Williams H, Blazeby JM (2017). Informing efficient randomised controlled trials: exploration of challenges in developing progression criteria for internal pilot studies. BMJ Open.

[73] Lewis JR (2018). The system usability scale: past, present, and future. Int J Hum Comput Interact.

[74] Heimgartner N, Meier S, Grolimund S, Ponti S, Arpagaus S, Kappeler F, Gaab J (2021). Randomized controlled evaluation of the psychophysiological effects of social support stress management in healthy women. PLoS One.

[75] Berger T, Boettcher J, Caspar F (2014). Internet-based guided self-help for several anxiety disorders: a randomized controlled trial comparing a tailored with a standardized disorder-specific approach. Psychotherapy (Chic).

[76] Schlicker S, Baumeister H, Buntrock C, Sander L, Paganini S, Lin J, Berking M, Lehr D, Ebert DD (2020). A Web- and mobile-based intervention for comorbid, recurrent depression in patients with chronic back pain on sick leave (Get.Back): pilot randomized controlled trial on feasibility, user satisfaction, and effectiveness. JMIR Ment Health.

[77] Kählke F, Berger T, Schulz A, Baumeister H, Berking M, Auerbach RP, Bruffaerts R, Cuijpers P, Kessler RC, Ebert DD (2019). Efficacy of an unguided internet-based self-help intervention for social anxiety disorder in university students: a randomized controlled trial. Int J Methods Psychiatr Res.

[78] Papadatou-Pastou M, Campbell-Thompson L, Barley E, Haddad M, Lafarge C, McKeown E, Simeonov L, Tzotzoli P (2019). Exploring the feasibility and acceptability of the contents, design, and functionalities of an online intervention promoting mental health, wellbeing, and study skills in higher education students. Int J Ment Health Syst.

[79] Levin ME, Pistorello J, Seeley JR, Hayes SC (2014). Feasibility of a prototype web-based acceptance and commitment therapy prevention program for college students. J Am Coll Health.

[80] Rose RD, Buckey JC Jr, Zbozinek TD, Motivala SJ, Glenn DE, Cartreine JA, Craske MG (2013). A randomized controlled trial of a self-guided, multimedia, stress management and resilience training program. Behav Res Ther.

[81] Melville KM, Casey LM, Kavanagh DJ (2010). Dropout from Internet-based treatment for psychological disorders. Br J Clin Psychol.

[82] Harrer M, Adam SH, Fleischmann RJ, Baumeister H, Auerbach R, Bruffaerts R, Cuijpers P, Kessler RC, Berking M, Lehr D, Ebert DD (2018). Effectiveness of an internet- and app-based intervention for college students with elevated stress: randomized controlled trial. J Med Internet Res.

[83] Kim S, Lee H, Kim H, Noh D, Lee H (2016). Effects of an integrated stress management program (ISMP) for psychologically distressed students: a randomized controlled trial. Perspect Psychiatr Care.

[84] Liverpool S, Mota CP, Sales CM, Čuš A, Carletto S, Hancheva C, Sousa S, Cerón SC, Moreno-Peral P, Pietrabissa G, Moltrecht B, Ulberg R, Ferreira N, Edbrooke-Childs J (2020). Engaging children and young people in digital mental health interventions: systematic review of modes of delivery, facilitators, and barriers. J Med Internet Res.

[85] Beatty L, Binnion C (2016). A systematic review of predictors of, and reasons for, adherence to online psychological interventions. Int J Behav Med.

[86] Nahum-Shani I, Shaw SD, Carpenter SM, Murphy SA, Yoon C (2022). Engagement in digital interventions. Am Psychol.

[87] Ritterband LM, Thorndike FP, Cox DJ, Kovatchev BP, Gonder-Frederick LA (2009). A behavior change model for internet interventions. Ann Behav Med.

[88] Alfonsson S, Olsson E, Hursti T (2016). Motivation and treatment credibility predicts dropout, treatment adherence, and clinical outcomes in an internet-based cognitive behavioral relaxation program: a randomized controlled trial. J Med Internet Res.

[89] Fernandez E, Salem D, Swift JK, Ramtahal N (2015). Meta-analysis of dropout from cognitive behavioral therapy: magnitude, timing, and moderators. J Consult Clin Psychol.

[90] Norcross JC, Krebs PM, Prochaska JO (2011). Stages of change. J Clin Psychol.

[91] Orji R, Vassileva J, Mandryk R (2012). Towards an effective health interventions design: an extension of the health belief model. Online J Public Health Inform.

[92] Ryan C, Bergin M, Wells JS (2018). Theoretical perspectives of adherence to web-based interventions: a scoping review. Int J Behav Med.

[93] Waller R, Gilbody S (2009). Barriers to the uptake of computerized cognitive behavioural therapy: a systematic review of the quantitative and qualitative evidence. Psychol Med.

[94] Rodriguez LM, Tomkins MM, Garey L, Young CM, Neighbors C (2022). Design, efficacy, and methodology considerations for brief interventions: Intervention delivery and incentives. Psychol Addict Behav.

[95] Winter N, Russell L, Ugalde A, White V, Livingston P (2022). Engagement strategies to improve adherence and retention in web-based mindfulness programs: systematic review. J Med Internet Res.

[96] Schleider JL, Weisz JR (2017). Little treatments, promising effects? Meta-analysis of single-session interventions for youth psychiatric problems. J Am Acad Child Adolesc Psychiatry.

[97] Schleider JL, Dobias ML, Sung JY, Mullarkey MC (2020). Future directions in single-session youth mental health interventions. J Clin Child Adolesc Psychol.

[98] Christensen H, Mackinnon A (2006). The law of attrition revisited. J Med Internet Res.

[99] Lee CS, Bowman M, Wu JL (2022). Preliminary outcomes from a single-session, asynchronous online, stress and anxiety management workshop for college students. Trends Psychiatry Psychother (Forthcoming).

[100] Van Daele T, Hermans D, Van Audenhove C, Van den Bergh O (2012). Stress reduction through psychoeducation: a meta- analytic review. Health Educ Behav.

[101] Rozental A, Boettcher J, Andersson G, Schmidt B, Carlbring P (2015). Negative effects of internet interventions: a qualitative content analysis of patients' experiences with treatments delivered online. Cogn Behav Ther.

[102] Kelders SM, Kok RN, Ossebaard HC, Van Gemert-Pijnen JE (2012). Persuasive system design does matter: a systematic review of adherence to web-based interventions. J Med Internet Res.

[103] Achilles MR, Anderson M, Li SH, Subotic-Kerry M, Parker B, O'Dea B (2020). Adherence to e-mental health among youth: considerations for intervention development and research design. Digit Health.

[104] Mohr DC, Schueller SM, Montague E, Burns MN, Rashidi P (2014). The behavioral intervention technology model: an integrated conceptual and technological framework for eHealth and mHealth interventions. J Med Internet Res.

[105] Ryan RM, Deci EL (2000). Self-determination theory and the facilitation of intrinsic motivation, social development, and well-being. American Psychologist.

[106] Ryan RM, Patrick H, Deci EL, Williams GC (2008). Facilitating health behaviour change and its maintenance: interventions based on Self-determination theory. Eur Health Psychol.

[107] Ali K, Farrer L, Gulliver A, Griffiths KM (2015). Online peer-to-peer support for young people with mental health problems: a systematic review. JMIR Ment Health.

[108] Horgan A, McCarthy G, Sweeney J (2013). An evaluation of an online peer support forum for university students with depressive symptoms. Arch Psychiatr Nurs.

[109] Kirsch DJ, Pinder-Amaker SL, Morse C, Ellison ML, Doerfler LA, Riba MB (2014). Population-based initiatives in college mental health: students helping students to overcome obstacles. Curr Psychiatry Rep.

[110] von Keyserlingk L, Yamaguchi-Pedroza K, Arum R, Eccles JS (2022). Stress of university students before and after campus closure in response to COVID-19. J Community Psychol.

[111] Wang C, Wen W, Zhang H, Ni J, Jiang J, Cheng Y, Zhou M, Ye L, Feng Z, Ge Z, Luo H, Wang M, Zhang X, Liu W (2021). Anxiety, depression, and stress prevalence among college students during the COVID-19 pandemic: a systematic review and meta-analysis. J Am Coll Health (Forthcoming).

[112] Asplund RP, Jäderlind A, Björk IH, Ljótsson B, Carlbring P, Andersson G (2019). Experiences of internet-delivered and work-focused cognitive behavioral therapy for stress: a qualitative study. Internet Interv.

[113] Irish M, Zeiler M, Kuso S, Musiat P, Potterton R, Wagner G, Karwautz A, Waldherr K, Schmidt U (2021). Students' perceptions of an online mental health intervention: a qualitative interview study. Neuropsychiatr.

[114] Jardine J, Earley C, Richards D, Timulak L, Palacios JE, Duffy D, Tierney K, Doherty G (2020). The experience of guided online therapy: a longitudinal, qualitative analysis of client feedback in a naturalistic RCT. Proceedings of the 2020 CHI Conference on Human Factors in Computing Systems.

[115] Berger T, Caspar F, Richardson R, Kneubühler B, Sutter D, Andersson G (2011). Internet-based treatment of social phobia: a randomized controlled trial comparing unguided with two types of guided self-help. Behav Res Ther.

[116] Zarski A, Lehr D, Berking M, Riper H, Cuijpers P, Ebert DD (2016). Adherence to internet-based mobile-supported stress management: a pooled analysis of individual participant data from three randomized controlled trials. J Med Internet Res.

[117] Cornish PA, Berry G, Benton S, Barros-Gomes P, Johnson D, Ginsburg R, Whelan B, Fawcett E, Romano V (2017). Meeting the mental health needs of today's college student: reinventing services through Stepped Care 2.0. Psychol Serv.

