# Supplementary material for: A Guided, Internet-Based Stress Management Intervention for University Students With High Levels of Stress: Feasibility and Acceptability Study
Source: JMIR Form Res. 2023 Nov 10;7:e45725. doi: 10.2196/45725 (PMC10674149; doi:10.2196/45725)
Supplement: Multimedia Appendix 1 [file formative_v7i1e45725_app1.pdf]

Multimedia Appendix 1

Changes to the Protocol

There are digressions from the protocol paper and the implementation. Below, differences were reported.

1. Secondary outcomes: We also applied the short version of the PSS-4 to assess weekly perceived stress. However, we did not use them in the analysis due to limited data.
2. Semi-structured interviews: During the planning of the study, we aimed to reach students who completed all sessions of the intervention for semi-structured interviews to understand their experience with the intervention in detail. Initially, we aimed to select participants based on the maximum variation sampling based on the different scores on the CSQ-8, SUS-10, and PSS-10. We tried to reach students who completed all sessions with varying scores, however, only a small number of students responded positively to the invitation for a semi-structured interview. Therefore we conducted interviews with the students who completed the core sessions of the intervention regardless of post-test assessment scores. During the process, we also expanded our focus by inviting non-completers to the semi-structured interviews, as they can help us understand adherence to a wider extent.
3. Core sessions involving first three sessions of the intervention are not defined in the protocol study. Previous studies highlighted the importance of defining core components of an intervention. Therefore we defined the the core sessions as the first three sessions involving psychoeducation, coping skills and cognitive restructuring , since these sessions provide the information and skills for symptom change based on the theoretical background of the intervention.
4. Upon reevaluating adherence as a secondary outcome and recognizing its direct and close association with the intervention's feasibility, we decided to reclassify adherence as a primary outcome in this study.
