# Supplementary material for: A Guided, Internet-Based Stress Management Intervention for University Students With High Levels of Stress: Feasibility and Acceptability Study
Source: JMIR Form Res. 2023 Nov 10;7:e45725. doi: 10.2196/45725 (PMC10674149; doi:10.2196/45725)
Supplement: Multimedia Appendix 3 [file formative_v7i1e45725_app3.pdf]

Multimedia Appendix 3

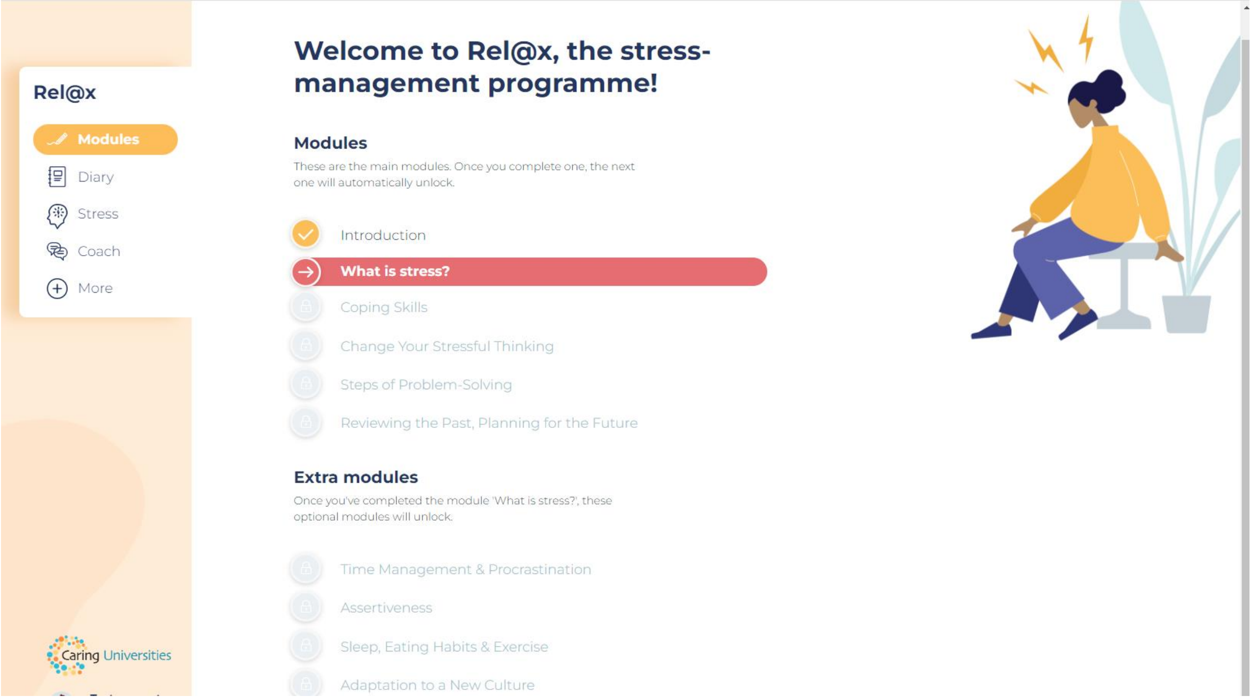

Stress Management Program

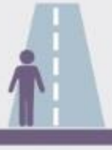

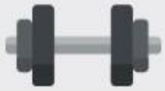

Session 1: What is stress?

Nature of the stress and how it affects you

Session 2: Coping Skills

Problem-based and emotion based coping skills

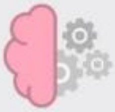

Session 3: Change the way of thinking

Dysfunctional thoughts and how to change them

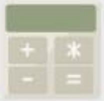

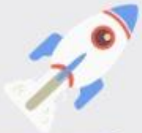

Session 4: Problem Solving

5 steps to solve problems

Session 5: Planning the Future

From past to the future: tips, plans

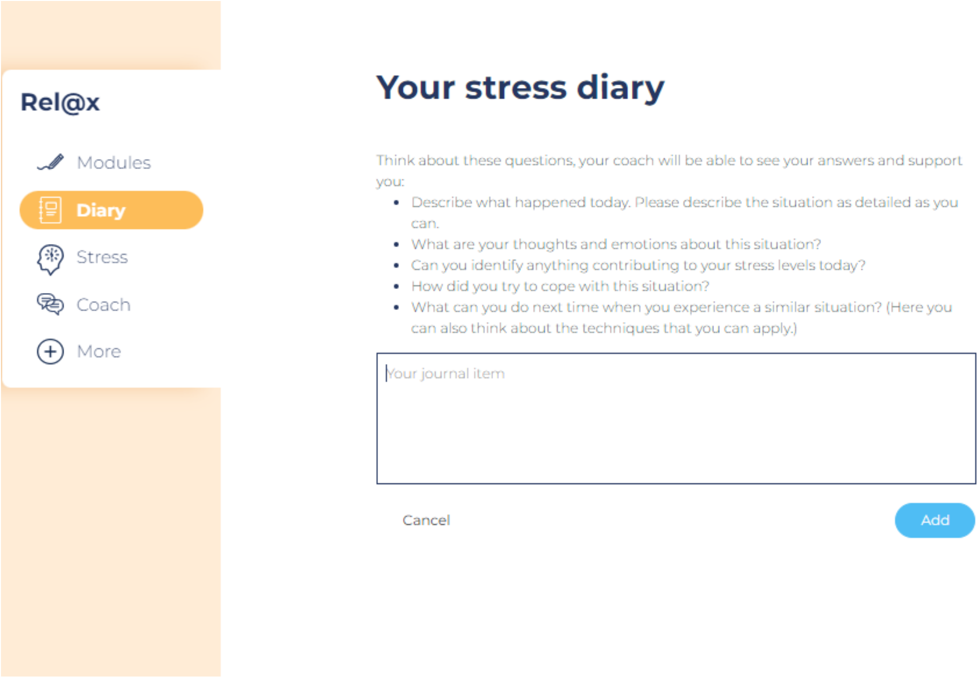
Rel@x
Modules
Diary
Stress
Coach
More
Your stress diary
Think about these questions, your coach will be able to see your answers and support you:
• Describe what happened today. Please describe the situation as detailed as you can.
• What are your thoughts and emotions about this situation?
• Can you identify anything contributing to your stress levels today?
• How did you try to cope with this situation?
• What can you do next time when you experience a similar situation? (Here you can also think about the techniques that you can apply.)
Your journal item
Cancel
Add

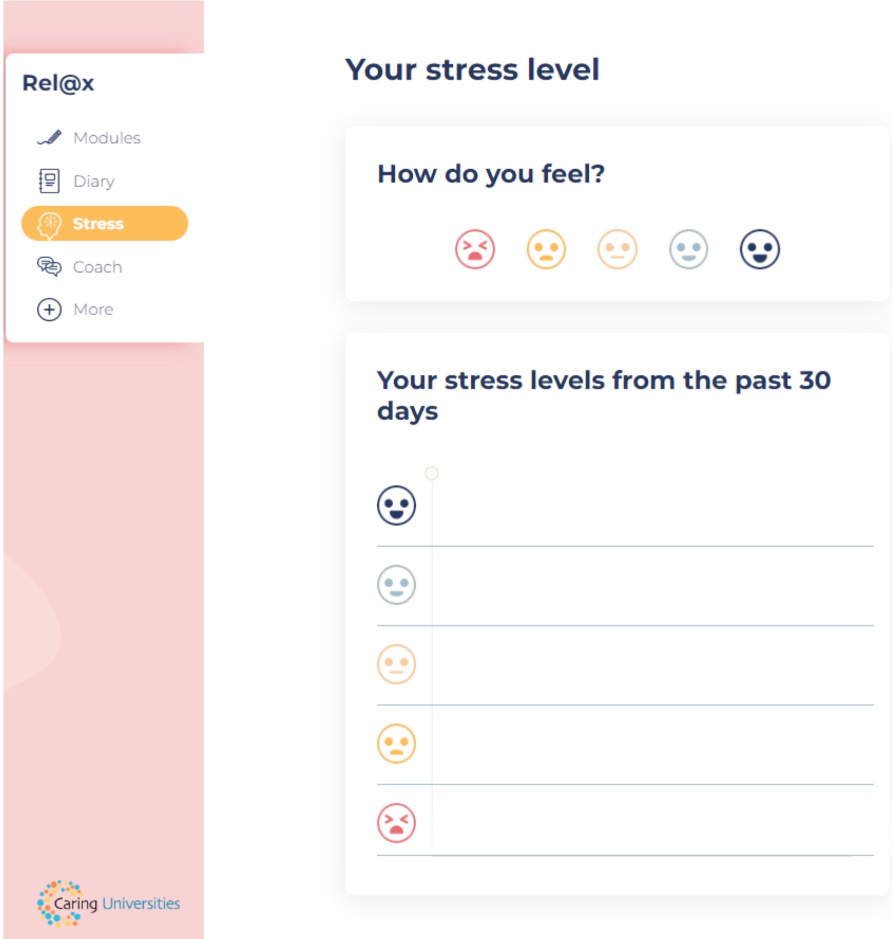
Rel@x
Modules
Diary
Stress
Coach
More
Caring Universities
Your stress level
How do you feel?
Your stress levels from the past 30 days
