# Supplementary material for: A Guided, Internet-Based Stress Management Intervention for University Students With High Levels of Stress: Feasibility and Acceptability Study
Source: JMIR Form Res. 2023 Nov 10;7:e45725. doi: 10.2196/45725 (PMC10674149; doi:10.2196/45725)
Supplement: Multimedia Appendix 4 [file formative_v7i1e45725_app4.pdf]

Multimedia Appendix 4

Questions regarding session evaluation

1) Were the goals of this Internet session clearly defined?
2) Was the content in this session clear and easy to understand?
3) Was this session easy to navigate?
4) Was the length of this session appropriate to the topic?
5) Were the illustrated pictures in this session helpful?
6) Did you understand the language, idiom, and words used in this session?
7) Do you think the case examples given in this session were appropriate for university students?
8) What did you learn from this session?
9) Is there anything you would want to change in this session?
10) Is there anything you want to mention about this session?
