# Supplementary material for: A Guided, Internet-Based Stress Management Intervention for University Students With High Levels of Stress: Feasibility and Acceptability Study
Source: JMIR Form Res. 2023 Nov 10;7:e45725. doi: 10.2196/45725 (PMC10674149; doi:10.2196/45725)
Supplement: Multimedia Appendix 5 [file formative_v7i1e45725_app5.pdf]

Multimedia Appendix 5

Survey questions for noncompleters

What was your reason for discontinuing the intervention? You can choose multiple items below.

Personal reasons

- ☐ I have no time.
- ☐ I have a high workload.
- ☐ My symptoms have improved to a point where I felt the intervention was not necessary anymore.
- ☐ I am experiencing some stressful life events (e.g. death of someone).
- ☐ I have found another source of help (e.g. psychotherapy, other self-help tools)

Intervention-related reasons

- ☐ The program is boring.
- ☐ The program is not user-friendly.
- ☐ The program is too demanding (too much text/exercises).
- ☐ The program is complicated.
- ☐ The program does not include the information that I need.
- ☐ I have no stable internet connection/ device.
- ☐ I do not feel comfortable with the degree of anonymity.
- ☐ I prefer face-to-face help.

E-coach-related reasons

- ☐ I feel that my e-coach does not support me.
- ☐ My goals do not fit with my e-coach`s goals.

Other:

Please specify your reason: _______________________
