# Supplementary material for: A Guided, Internet-Based Stress Management Intervention for University Students With High Levels of Stress: Feasibility and Acceptability Study
Source: JMIR Form Res. 2023 Nov 10;7:e45725. doi: 10.2196/45725 (PMC10674149; doi:10.2196/45725)
Supplement: Multimedia Appendix 6 [file formative_v7i1e45725_app6.pdf]

Multimedia Appendix 6

Questions for semistructured interviews (for completers)

1) What were your initial thoughts and feelings regarding this Internet-based stress management program?
2) How was your overall experience of using the program?
- *What are your general thoughts and opinions on the platform? (navigation, structuring, how it looks)*
3) Generally, how did you feel about this program?
4) Do you feel anything has changed in your life since using the program?
5) To what extent did you find it convenient for your needs/ preferences?
6) What was your experience with the program being online and delivered via the internet?
- What were your reasons that you prefer this program as an internet-based treatment over face-to-face support?
8) How did the program fit in with your lifestyle?
9) Were there any enjoyable parts in the program? (And why?)
- Were there any parts that you did not enjoy/ useful?
10) Is there anything you would want to change or add to this program?
11) What are your general thoughts and opinions on the coaching you received? (content, frequency)

Questions for semistructured interviews (for non-completers)

1. How did you hear about this program?
2. What were your expectations about this program at the beginning?
3. How did you find the program being online and delivered via the internet?
4. What were your reasons that you prefer this program as an internet-based treatment over face-to-face support?
5. What were your initial thoughts and feelings regarding this Internet-based stress management program?
6. How was your overall experience doing the program?
7. What was your reason for discontinuing the program?
8. What did you like about it?
9. What did you dislike about it?
10. Were there any enjoyable/useful parts in the program? (And why?)
11. Were there any parts that you did not enjoy?
12. What would make this program more attractive?
13. What would keep you to continue to the program?
14. Is there anything you would want to change or add to this program?
