# Supplementary material for: A Guided, Internet-Based Stress Management Intervention for University Students With High Levels of Stress: Feasibility and Acceptability Study
Source: JMIR Form Res. 2023 Nov 10;7:e45725. doi: 10.2196/45725 (PMC10674149; doi:10.2196/45725)
Supplement: Multimedia Appendix 7 [file formative_v7i1e45725_app7.pdf]

Multimedia Appendix 7

Baseline characteristics of the participants

		Total sample (N=436)	Study			Intervention		
			Noncompleters (n=286)	Completers (n=150)	*P* value	Noncompleters (n=333)	Completers (n=103)	*P* value
Age (years), mean (SD)[a]		22.89 (0.17)	23 (3.49)	22.70 (3.9)	.42	22.97 (3.8)	22.65 (3.05)	.44
**Sex[b], n (%)**					.27			.20
	Female	382 (87.6)	247 (86.4)	135 (90)		288 (86.5)	94 (91.3)	
	Male	54 (12.3)	39 (13.6)	15 (10)		45 (13.5)	9 (8.7)	
**Nationality[b], n (%)**					.36			.44
	Dutch	272 (62.4)	175 (61.2)	97 (64.7)		205 (61.6)	67 (65)	
	European but not Dutch	136 (31.2)	88 (30.8)	48 (32)		103 (30.9)	33 (32)	
	North American	4 (0.9)	4 (1.4)	0 (0)		4 (1.2)	0 (0)	
	South American	6 (1.4)	5 (1.7)	1 (0.7)		6 (1.8)	0 (0)	
	Asian	18 (4.1)	14 (4.9)	4 (2.7)		15 (4.5)	3 (2.9)	
**University[b], n (%)**					.08			.03
	Leiden University	121 (27.8)	79 (27.6)	42 (28)		90 (27)	31 (30.1)	

	Maastricht University	77 (17.7)	43 (15)	34 (22.7)		52 (15.6)	25 (24.3)	
	Erasmus University Rotterdam	8 (1.8)	7 (2.4)	1 (0.7)		8 (2.4)	0 (0)	
	Utrecht University	166 (38.1)	108 (37.8)	58 (38.7)		127 (38.1)	39 (37.9)	
	Vrije University Amsterdam	64 (14.7)	49 (17.1)	15 (10)		56 (16.8)	8 (7.8)	
**Education level[b], n (%)**					.56			.49
	First year	70 (16.1)	42 (14.7)	28 (18.7)		55 (16.5)	15 (14.6)	
	Second year	54 (12.4)	34 (11.9)	20 (13.3)		43 (12.9)	11 (10.7)	
	Third year	73 (16.7)	49 (17.1)	24 (16)		52 (15.6)	21 (20.4)	
	Fourth year	26 (5.9)	14 (4.9)	12 (8)		17 (5.1)	9 (8.7)	
	Master's degree student	203 (46.6)	140 (48.9)	63 (42)		157 (47.1)	46 (44.7)	
	PhD student	10 (2.3)	7 (2.4)	3 (2)		9 (2.7)	1 (0.9)	
**Marital status[b], n (%)**					.56			.45
	Single	229 (52.5)	161 (56.3)	68 (45.3)		181 (54.4)	48 (46.6)	
	In a relationship	186 (42.7)	112 (39.2)	74 (49.3)		135 (40.5)	51 (49.5)	
	Married	16 (3.7)	10 (3.5)	6 (4)		13 (3.9)	3 (2.9)	

	Other	5 (1.1)	3 (1)	2 (1.3)		4 (1.2)	1 (0.9)	
**Current professional help[b], n (%)**					.11			.06
	Medication	15 (3.4)	11 (3.8)	4 (2.7)		14 (4.2)	1 (0.9)	
	Psychotherapy or counseling	43 (9.9)	31 (10.8)	12 (8)		34 (10.2)	9 (8.7)	
	Both	9 (2.1)	8 (2.8)	1 (0.7)		7 (2.1)	2 (1.9)	
	None	367 (84.2)	236 (82.5)	131 (87.3)		278 (83.5)	89 (86.4)	
	Missing	2 (0.5)	0 (0)	2 (1.3)		0 (0)	2 (1.9)	
**Baseline clinical characteristics, mean (SD)**								
	PSS-10[a,c]	25.45 (3.68)	25.56 (3.73)	25.23 (3.59)	.38	25.48 (3.72)	25.35 (3.57)	.76
	PHQ-9[a,d]	10.39 (4.18)	10.62 (4.31)	9.95 (3.88)	.11	10.67 (4.25)	9.48 (3.80)	.011
**Depressive symptoms severity, n (%)**					.50			.11
	None to minimal	29 (6.7)	19 (6.6)	10 (6.7)		18 (5.4)	11 (10.7)	
	Mild	169 (38.8)	107 (37.4)	62 (41.3)		125 (37.5)	44 (42.7)	
	Moderate	157 (36)	101 (35.3)	56 (37.3)		123 (36.9)	34 (33)	
	Moderately severe	81 (18.6)	59 (20.6)	22 (14.7)		67 (20.1)	14 (13.6)	
EQ-5D-5L index[e], mean (SD)		0.71 (0.16)	0.67 (0.17)	0.71 (0.13)	.28	0.69 (0.17)	0.73 (0.12)	.56
EQ-5D-5L VAS[a], mean (SD)		57.92 (19.17)	57.16 (19.25)	59.37 (18.99)	.25	57.86 (19.27)	58.13 (18.94)	.90

[a]2-tailed Independent sample *t* test.

[b]Chi-square test.

[c]PSS-10: Perceived Stress Scale–10.

[d]PHQ-9: Patient Health Questionnaire–9.

[e]Mann-Whitney *U* test.
