# Supplementary material for: A Guided, Internet-Based Stress Management Intervention for University Students With High Levels of Stress: Feasibility and Acceptability Study
Source: JMIR Form Res. 2023 Nov 10;7:e45725. doi: 10.2196/45725 (PMC10674149; doi:10.2196/45725)
Supplement: Multimedia Appendix 8 [file formative_v7i1e45725_app8.pdf]

Multimedia Appendix 8

Tables for additional outcomes

Table S1. Findings of the working alliance for the total number of participants, and separately for each session completers

	**Total (n = 135)**	**Intervention completers (n = 75)**	**Non-completers (n = 60)**	***p***
	M (SD)			
WAI-12	42.82 (8.49)	46.37 (6.73)	38.38(8.42)	0.000
WAI-bond	15.33 (3.82)	16.32 (3.41)	14.11 (3.96)	0.001
WAI-goal and tasks	27.53 (6.03)	30.05 (4.60)	24.37 (6.14)	0.000

Table S2. Rates on the usefulness of each session

	Total			Intervention completers			Non-completers		
	N	*M*	*(SD)*	N	*M*	*(SD)*	N	*M*	*(SD)*
Introduction	228	75.18	19.25	64	82.06	13.91	164	72.50	20.38
Session 1: Psychoeducation about stress	180	74.93	17.76	65	80.35	13.64	115	71.87	19.09
Session 2: Coping skills and emotion regulation	118	78.64	16.57	65	83.89	13.04	53	72.19	18.21
Session 3: Cognitive restructuring	80	82.83	12.20	66	82.38	12.65	14	84.93	9.93
Session 4: Problem solving strategies	51	74.92	20.66	51	74.92	20.66	-	-	-
Session 5: Reviewing the stress responses, and setting goals for the future	39	79.74	14.92	39	79.74	14.92	-	-	-
Optional Session 1: Assertiveness	22	67.86	20.31	17	65.76	19.93	5	75.00	22.260
Optional Session 2: Adaptation to a new culture	8	71.13	18.64	7	67	15.70	1	100	
Optional Session 3: Time management	39	73.18	16.68	25	71.52	17.29	14	76.14	15.69

and procrastination									
Optional Session 4: Sleep, Eating, and Exercise	33	66.06	21.36	22	65	21.04	11	68.18	22.86

Table S3. Evaluations of each main session for the whole sample

	Introduction		Session 1: Psychoeducation about stress		Session 2: Coping skills and emotion regulation		Session 3: Cognitive restructuring		Session 4: Problem-solving strategies		Session 5: Reviewing the stress responses, and setting goals for the future	
	n	%	*n*	%	*n*	%	*n*	%	*n*	%	*n*	%
Were the goals of this Internet session clearly defined?	270	90.6	209	90.1	131	87.9	100	98	65	97	47	94
Was the content in this session clear and easy to understand?	295	99.0	221	95.3	142	95.3	98	96.1	65	97	50	100
Was this session easy to navigate?	292	98.6	225	97.8	147	98.7	98	96.1	65	98.5	50	100
Was the length of this session appropriate to the topic?	273	91.6	192	83.5	121	81.2	91	89.2	61	91	44	88
Were the illustrated pictures in this session helpful?	215	72.1	153	66.2	119	80.4	91	89.2	59	88.1	42	84
Did you understand the language, idiom, and words used in this session?	294	99.3	222	96.1	144	97.3	99	98	65	97	50	100
Do you think the case examples given in this session were appropriate for university students?	247	83.2	191	82.7	131	89.1	95	93.1	60	89.6	45	90

Table S4. Evaluations of optional sessions for the whole sample

	Optional Session 1: Assertiveness		Optional Session 2: Adaptation to a new culture		Optional Session 3: Time management and procrastination		Optional Session 4: Sleep, Eating, and Exercise	
	N	%	N	%	N	%	N	%
Were the goals of this Internet session clearly defined?	24	75	8	100	45	87	33	75
Was the content in this session clear and easy to understand?	30	94	8	100	50	96	43	98
Was this session easy to navigate?	31	97	8	100	50	96	43	98
Was the length of this session appropriate to the topic?	17	53	8	100	35	69	30	68
Were the illustrated pictures in this session helpful?	24	75	7	87.5	45	87	36	82
Did you understand the language, idiom, and words used in this session?	31	100	8	100	52	100	42	95
Do you think the case examples given in this session were appropriate for university students?	27	84	8	100	42	81	32	73

Table S5. Dropout Reasons (N= 42)

Personal reasons	n	%
No time	18	42.9
Lost interest/ Motivation	23	54.76
Symptoms improved	14	33.3
Experiencing stressful event	5	11.9
Another source of help	13	31
Intervention related reasons		
Boring	4	9.5
Difficult navigation	-	-
Too demanding	6	14.3
Too complicated	-	-
Not including needed information	9	21.4
No stable internet connection	-	-
Feeling uncomfortable with the degree of anonymity	2	4.8
Preference for face-to-face help	13	31
Feeling not supported by eCoach	5	11.9
Different goals with eCoach	2	4.8
