# Supplementary material for: A Guided, Internet-Based Stress Management Intervention for University Students With High Levels of Stress: Feasibility and Acceptability Study
Source: JMIR Form Res. 2023 Nov 10;7:e45725. doi: 10.2196/45725 (PMC10674149; doi:10.2196/45725)
Supplement: Multimedia Appendix 9 [file formative_v7i1e45725_app9.pdf]

Multimedia Appendix 9

Extended Results of the Semi-Structured Interview

As result of qualitative analysis, we generated five themes: 1) considerations of initiating the intervention, 2) intervention-related factors influencing user experience, 3) appraisals of experienced support by the eCoach, 4) personal factors interfering with the user experience, and 5) suggestions for improvement of the intervention. All themes and subthemes, including the participants, were provided in Table S1.

Theme 1: Considerations of initiating the intervention

This theme describes the pre-intervention experiences of the students initiating the intervention. In this theme, we classified the responses under three subthemes, namely perceived need, expectations towards the intervention, and initial perceptions of the intervention format.

Subtheme 1: Perceived need

It describes the internal (e.g. experiencing a stressful period or elevated stress response) and external factors (e.g. reinforcement of the student psychologist's advice) associated with the increased perceived need. Several participants highlighted the importance of timing to initiate the intervention by expressing their increased perceived need for receiving help such as experiencing a stressful period, or elevated levels of stress responses. Some participants also reported that external factors facilitated the initiation such as the advice of a student psychologist or the relevance of the feedback as a result of screening.

Subtheme 2: Expectations towards the intervention

This subtheme refers to the beliefs, emotions, and attitudes towards the benefits of intervention and research. Although some participants reported positive expectations towards the benefit of the intervention or positive emotions such as curiosity and excitement, many participants started the intervention with unclear expectations.

Subtheme 3: Initial perceptions of the intervention format

It refers to beliefs, emotions, and attitudes towards the facilitators or barriers related to the intervention format. Most students highlighted lower barriers to reaching help as an important factor in starting the intervention. Another common reason was to be able to work with an eCoach in addition to the flexibility and suitability of the intervention with the COVID-19 regulations.

Theme 2: Intervention-related factors influencing user experience

User experience was defined as an umbrella concept in our study, which refers to cognitive, emotional, and behavioral responses related to the intervention. This theme covers the different aspects of user experience, including adherence, satisfaction, and usability of the intervention.

Subtheme 1: Attractiveness of user interface

This refers to the *"look and feel"* of the intervention [55], including aesthetic elements of the intervention (e.g., colors, graphic design, and multimedia) and clarity of the layout and navigation of the intervention. The majority of the participants found the intervention aesthetically appealing and straightforward. However, few participants reported different preferences related to layout, such as choices for more minimalistic graphs and pictures, while one participant reported a preference for drawings instead of photos in the intervention.

Subtheme 2: Usability of the intervention and its features

This subtheme refers to users` assessment of the ease of use and required effort to follow intervention and its features, including duration, frequency, intensity, modality, and other technical aspects. Most of the students who completed the intervention perceived the intervention as not too demanding. However, the majority of the non-completer students reported that the intervention required high demand. Most students shared the positive experience with the flexibility of the intervention regarding fitting their schedule, being able to arrange the pace and opportunities for re-reading the content when needed and having a space to think and reflect. However, some students reported flexibility challenges, such as

feeling less pressure to follow the intervention and thus feeling less committed and easily postponing the intervention. Some students also expressed their need for face-to-face interaction; even one student reported discontinuation because of screen fatigue. Some students also reported difficulties using some intervention features such as the stress diary. Although several students reported benefits of a stress diary, others reported dissatisfaction with doing activities requiring regular or immediate access to the website.

Subtheme 3: Relevance of the content

This subtheme describes the extent to which students assess information and topics covered in the intervention as suitable, relevant, and credible. Students generally showed high satisfaction with the content by learning new aspects of stress and coping, and the relevance of the topics and examples to college life. However, some students reported a lack of novelty or lack of hands-on aspects such as practical activities as the downsides of the intervention.

Subtheme 4: Convenience of the tailoring and personalization

It represents the students` responses related to the customization of the content based on their needs and preferences. Although many students found the intervention as tailored to the college students, and shared their positive experiences with customizable aspects of the intervention (e.g. eCoach selection, optional sessions, and personalized feedback by the eCoach), some students reported their preferences for increasing personalization by enhanced optional content and increasing the communication with eCoach.

Subtheme 5: Helpfulness of the intervention

This subtheme refers to the perceived impact of the intervention on symptom improvement, acquiring new skills, and increasing awareness. Several students who completed the intervention reported positive effects of the intervention for stress-related symptoms. Some students also reported perceived decreased symptomatology since the beginning of the intervention, however, they stated uncertainty about whether improvements were attributable to the effect of the intervention, as they reported changes in the stressful situations. Other than symptom-related outcomes, some students also mentioned the

positive effect of an online intervention to seek further formal help for psychological problems.

Theme 3: Appraisals of experienced support by the eCoach

This represents the students` experiences with the received support. The central focus of this theme is to what extent and in what way they felt supported specifically by the eCoach.

Subtheme 1: Perceived social or emotional support from the eCoach

Most of the students reported that they felt understood and empathy by the eCoach. One student also reported feeling safe while working with eCoach.

Subtheme 2: Perceived informational support from the eCoach

Some students shared positive remarks and appreciation for the eCoach`s feedback on their progress and guidance. However, few students also addressed the need for deeper communication with the eCoach by changing the frequency of the interaction, preferences for face-to-face or synchronous communication with the eCoach, or frustrations with the content of the feedback.

Subtheme 3: Skepticism toward professionality of the peer eCoach

Few students, especially those who did not continue the intervention, shared their concerns and reservations about the eCoach`s competence and qualifications. Working with a peer seems to be a drawback for some students, as they mentioned preferences for more experienced mental health professionals instead of peer-delivered help.

Theme 4: Personal factors interfering with the user experience

This theme includes the personal aspects that might affect intervention experiences.

Subtheme 1: Busy schedule

Some students reported that whether they had a busy schedule or not affected their intervention use. Having a busy schedule and not prioritizing the intervention among other academic or daily tasks was an impeding factor in using the intervention.

Subtheme 2: Symptom severity and changing needs

Similar to the first theme about considerations of initiation of using the intervention, students` perceived needs continued to play a role in the continuation of the intervention. One student reported deterioration and perceived need to work with a professional instead of following an online intervention as a reason for discontinuation. On the contrary, lack of perceived need was mentioned as a reason for some participants. Some students reported change in the stressful situation (e.g. conflict with the supervisor but then changing the supervisor) as an influencing personal factor interfering with the intervention use.

Subtheme 3: Changing motivation

Some students reported that motivation was an important factor playing role in the user experience. As many students started the intervention with increased motivation, during the procedure, some students could sustain their motivation, while others became demotivated which, reportedly, can be a factor in the commitment to the intervention. For example, one student who completed the intervention described herself as a highly motivated person for self-care and attributed this characteristic as a possible factor to continue the intervention. Similarly, some students perceived lowering motivation as a barrier to a positive user experience. Moreover, some students also marked difficulties staying motivated to continue the intervention especially when they were having elevated stress responses.

Theme 5: Suggestions for improvement of the intervention

This theme was generated based on the students` recommendations for optimizing the intervention and enhancing the user experience. We classified the recommendations under four subthemes to make them in line with the abovementioned themes, as they have overlapping aspects.

Subtheme 1: Enhancing the attractiveness of user interface and usability

The suggestions regarding the intervention interface and ease of use were collated under this subtheme. It turned out that although most of the students were satisfied with the user interface, varying preferences and needs exist regarding the wishes for different layouts, integrating several features such as using mobile phone application as an alternative delivery method, provision of downloadable documents, and including more multimedia sources and adjustable length of the sessions. One student also preferred to be able to self-select all sessions instead of structured intervention. Another student also recommended adding an estimated time for completion of each activity in the intervention to increase predictability.

Subtheme 2: Optimizing the intervention content

This theme includes recommendations for the refinement of the intervention content to make it more relevant. Our findings showed that students generally reported satisfaction with the content, however, some additions could improve the intervention for example diversifying the content of the session by offering diverse optional sessions, integrating time and mood-dependent exercise, improving the novelty of the content, embedding relaxation and meditation techniques into the intervention. Some students also reported the need for diversifying the topics and providing more in-depth stories of the fictitious characters in the intervention.

Subtheme 3: Improving support and interaction in the intervention

This subtheme represents the proposed changes to improve support and interaction in the platform. We found that some students reported a high need for social interaction on the platform. Some students suggested adding a forum in which other students can exchange ideas for stress management and share their experiences with stress. Another major recommendation was to increase the interaction with the eCoach through synchronous or more frequent communication or even the arrangement of a face-to-face meeting(s) at least at some point in the intervention. Involving mental health professionals was also a further suggestion. Some students reported the need to work with a mental health professional at certain points, for example choosing the best fitting intervention based on the symptoms

after screening or maybe at the end of the intervention in case no improvement or deterioration occurs.

Subtheme 4: Improving implementation and integration

A few suggestions were also provided for the promotion and implementation of the intervention. Some students reported perceived support from the university, just by offering psychological interventions for college students. In addition, some students recommended placing the intervention in a context where other services at the university are integrated. One student suggested developing strategies to facilitate referral to on and off-campus services for students with different needs. Few students also mentioned the importance of the visibility of this intervention and highlighted the improving promotion of this intervention.

Table S1. Themes and subthemes from the semi-structured interviews

**Theme 1: Considerations of initiating the intervention**		
Sub-themes	Codes	Participants
Subtheme 1:		*C1, C2, C3, C6, C8, C10, C11, D6, D7*
Perceived need	Stressful timing to initiate the intervention	
	No stressful timing	*C4*
	External factors facilitating uptake	*C1, C3, C5, C7, C8, D3, D5*
Subtheme 2:	Positive feelings and expectations	*C1, C3, C6, C9, C11, C4, D1, D4*
Expectations towards the intervention		
	Openness towards intervention	*C2, C4, C5, C7, C8, C9, C10, C11, D2, D3 D5*
	Suitable for the pandemic situation	*C3, C5, D2, D4, D6*
Subtheme 3:	Low barrier and increased accessibility	*C1, C2, C4, C9, C10, D1, D2, D3, D6, C11*
Initial perception of the intervention format	Flexibility	*C4, C11, D1, D2, D5, D6*
	Getting used to online contact	*D6*
	Opportunity to work with an eCoach	*C4, C5, C6, C9, D2, D3, D4*
**Theme 2: Intervention-related factors influencing user experience**		

Sub-themes	Codes	Participants
Subtheme 1: Attractiveness of user interface	Convenient visual design	*C1, C2, C3, C4, C5, C6, C8, C9, D1,D2, D5, D6*
	Preferences for different layout alternatives	*C11, D7*
Subtheme 2: Usability of the intervention and its features	Difficulty in some web-based features	*C1, C2, C3, C4, C5, C6, C7, C9, C10, C11, D7*
	Too demanding content	*C10, D1 D3, D4, D5*
	Not too demanding content	*C1, C2, C4, C6, C7, C8, C11,D2*
	Clarity of the sessions	*C1,C2, C3, C5, C6, C8, C10, C11, D7*
	Engaging/stimulating language	*C2,C5,* C6, *C7, C11, D1, D3, D6*
	Valuing anonymity	*C2, C4,*
	Need for synchronous communication	*C5, C6, C9, C10, C11, D2, D3, D5, D6, D7*
	Need for face-to-face interaction rather than an online format	*C6, C10, C11, D2, D5,D6*
	No missing the components of face-to-face help	*C1, C2*
	Screen fatigue	*D4*
	No impact of reminders	*D3*
	Positive impact of reminders	*C8, D6*
	Increased flexibility	*C1, C2, C3, C4, C7, C8, C10, C11, D5, D7*
	Challenges of too much flexibility and autonomy	*C4, C10, D2, D3, D5,*
	Feeling comfortable and creative	*C1, D3, C6, C7, C11*
	Good frequency of feedback from ecoach	*D3, D5*
	Need for more frequent feedback from ecoach	*C4, C9, C10, D6*
Subtheme 3: Relevance of the content	Familiarity	*C3, D2, D5*
	Perceived fit	*C10*

	Too theoretical, absence of practical components	*C4, D4*
	Too confrontational	*D1*
	Scientific information and high credibility	*C2, D3*
Subtheme 4:<br><br>Convenience of the tailoring and personalization	Modifying the content to personal preferences and selection of the e-coach	*C2, C4, C7, C8, 10, D2, D4, D7*
	Feeling more personalized because of eCoach`s feedback	*C5, C7, C8, C9, C11, D2, D3*
	Preferences for more personalized help	*D1, D2, D3, D4, D5*
Subtheme 5: Helpfulness of the intervention	Gaining insights and skills for immediate stress reduction and prevention of future stress-related problems	*C1, C2, C3, C5,C6, C7, C8, C10, C11, D1,D2, D4, D5, D7*
	Encouraging for the future professional help	*C2,C4, C5, D1*
	Unclear effects of the intervention on improving symptoms	*C4, C9, D3*
	The inconvenience of the intervention for more severe problems	*C8, D1, D6*
**Theme 3: Appraisals of experienced support by the eCoach**		
Sub-themes	Codes	Participants
Subtheme 1:<br><br>Perceived social or emotional support from the eCoach	Feeling heard by the eCoach	*C2, C3, C5, C8, C11, D1, D2, D4*
	Feeling safe with the eCoach	*C5*
Subtheme 2:	Appreciation of receiving feedback about their progress from the eCoach	*C1, C2, C4, C5, C6, C8, C9, C10, C11, D3, D4, D5*

Perceived informational support from the eCoach		
	Need for in-depth feedback from the eCoach	*C1, C4, C5, C9, C11, D1, D2, D5, D7*
Subtheme 3:<br><br>Skepticism toward professionality of the peer eCoach	Preference for a mental health professional over a peer eCoach//	*D1, D3, D4, D5*
Theme 4: Personal factors interfering with the user experience		
Sub-themes	Codes	Participants
Subtheme 1: Busy schedule	Busy schedule, problems related to time management	*D1, D2, D3, D7*
	Not very busy	*C2*
Subtheme 2: Symptom severity and changing needs	Worsening mental health state	*D6*
	Lack of perceived need	*D7*
Subtheme 3: Changing motivation	Lack of motivation due to experiencing high levels of stress	*D1, D3, D6*
	High/ Intrinsic motivation to follow the intervention	*C2, C3, C7, C10*
	Feeling not ready to change	*D1*
**Theme 5: Suggestions for improvement of the intervention**		
Sub-themes	Codes	Participants
Subtheme 1:<br><br>Enhancing the attractiveness of user interface and usability	Increasing flexibility through modifiable layout and adjustable sessions length	*C2, C6, C7, C10, D2, D3, D4*
	Integration of additional features	*C2, C5, C6, C11, D4, D6*
	Increasing predictability	*D4*

Subtheme 2: Optimizing the intervention content	Diversifying the content of the intervention	*C2, C3, C4, D2, D4, D6*
	Enhancing guidance through examples	*C2, C3, C9, D5, D6*
Subtheme 3: Improving support and interaction	Increasing *user-user* interaction	*C4, D1, D3, D4, D5*
	Increasing *user- eCoach* interaction	*C5, C9, C10, D2, D3, D5, D6, D7*
	Increasing intervention-user interaction	*D4*
	Involving mental health professionals	*D1, D4, D5*
Subtheme 4: Improving implementation and integration	Integration of the relevant on/ off campus services into the intervention	*C7, C9*
	Increasing visibility of the intervention	*C8, D7*
	Continuing provision of institutional support	*C2, D3, D6*
